# Associations between Variation in *CHRNA5-CHRNA3-CHRNB4*, Body Mass Index and Blood Pressure in the Northern Finland Birth Cohort 1966

**DOI:** 10.1371/journal.pone.0046557

**Published:** 2012-09-27

**Authors:** Marika Kaakinen, Francesca Ducci, Mikko J. Sillanpää, Esa Läärä, Marjo-Riitta Järvelin

**Affiliations:** 1 Institute of Health Sciences, University of Oulu, Oulu, Finland; 2 Biocenter Oulu, Oulu, Finland; 3 Psychological Medicine, Institute of Psychiatry, King's College, London, United Kingdom; 4 Division of Mental Health, St. George's University of London, London, United Kingdom; 5 Department of Mathematical Sciences, University of Oulu, Oulu, Finland; 6 Department of Biology, University of Oulu, Oulu, Finland; 7 Department of Life Course and Services, National Institute for Health and Welfare, Oulu, Finland; 8 Department of Epidemiology and Biostatistics, MRC-HPA Centre for Environment and Health, Imperial College London, London, United Kingdom; Vanderbilt University, United States of America

## Abstract

**Background:**

The *CHRNA5-CHRNA3-CHRNB4* gene cluster on 15q25 has consistently been associated with smoking quantity, nicotine dependence and lung cancer. Recent research also points towards its involvement in cardiovascular homeostasis, but studies in large human samples are lacking, especially on the role of the gene cluster in blood pressure regulation.

**Methodology/Principal Findings:**

We studied the associations between 18 single nucleotide polymorphisms (SNPs) in *CHRNA5-CHRNA3-CHRNB4* and systolic blood pressure (SBP), diastolic blood pressure (DBP), and body mass index (BMI) in 5402 young adults from the Northern Finland Birth Cohort 1966. We observed some evidence for associations between two SNPs and SBP and between six SNPs and BMI; the evidence for associations with DBP was weaker. The associations with the three phenotypes were driven by different loci with low linkage disequilibrium with each other. The associations appeared more pronounced in smokers, such that the smoking-increasing alleles would predict lower SBP and BMI. Each additional copy of the rs1948 G-allele and the rs950776 A-allele reduced SBP on average by −1.21 (95% CI −2.01, −0.40) mmHg in smokers. The variants associated with BMI included rs2036534, rs6495309, rs1996371, rs6495314, rs4887077 and rs11638372 and had an average effect size of −0.38 (−0.68, −0.08) kg/m^2^ per an additional copy of the risk allele in smokers. Formal assessments of interactions provided weaker support for these findings, especially after adjustment for multiple testing.

**Conclusions:**

Variation at 15q25 appears to interact with smoking status in influencing SBP and BMI. The genetic loci associated with SBP were in low linkage disequilibrium with those associated with BMI suggesting that the gene cluster might regulate SBP through biological mechanisms that partly differ from those regulating BMI. Further studies in larger samples are needed for more precise evaluation of the possible interactions, and to understand the mechanisms behind.

## Introduction

The *CHRNA5-CHRNA3-CHRNB4* gene cluster at 15q25 has consistently been associated with smoking quantity and nicotine dependence [1]–[15], as well as with lung cancer and chronic obstructive pulmonary disease [10], [13], [16]–[19]. The genes in the region encode the nicotinic acetylcholine receptor (nAChR) subunits α3, α5 and β4, of which α3 and β4, and possibly also α5 are expressed in autonomic ganglia [20]. Their presence in these sites suggests also a possible involvement in the regulation of autonomic function and thus, cardiovascular homeostasis.

Animal studies exploring the role of nAChRs in cardiovascular risk factors exist but they have mainly focused on nAChR subunits α4β2 and α7. These studies have demonstrated the involvement of these subunits in central cardiovascular actions, such as the regulation of blood pressure (BP) and heart rate [21]–[23]. In contrast, studies on the *CHRNA5-CHRNA3-CHRNB4* gene cluster and cardiovascular homeostasis in humans are scarce. Rana *et al.* observed in a twin study of 370 individuals that polymorphisms in the *CHRNA5-CHRNA3-CHRNB4* gene cluster were associated with systolic blood pressure (SBP), circulating levels of catestatin (an endogenous antagonist of nAChR) and epinephrine. These results suggest that variation in *CHRNA5-CHRNA3-CHRNB4* gene cluster, already implicated in smoking behavior, is also involved in the autonomic regulation of BP. The associations between variation in *CHRNA5-CHRNA3-CHRNB4* and SBP persisted after adjustment for smoking, suggesting an effect independent of smoking. [24] Another study on humans found that polymorphisms in *CHRNA3* increased susceptibility to develop peripheral arterial disease among smokers [13]. In addition, a large meta-analysis of over 24000 subjects demonstrated that *CHRNA3*-rs1051730 interacts with smoking status in influencing body mass index (BMI). The allele that predisposes to heavier smoking was associated with a lower BMI in those who smoked but not in non-smokers. [25]

The potential involvement of these subunits in cardiovascular homeostasis is intriguing and could at least partly explain associations between smoking and BP observed in previous epidemiological studies. Smoking has been associated with BP in several studies with smokers having on average a lower BP than non-smokers [26]. This association is often explained by the appetite-suppressing effect of smoking [27] or by smoking serving as a behavioral alternative to eating resulting in decreased food intake [28]. Both mechanisms would lead to a leaner body in smokers, and thus a lower BP. However, an alternative explanation could be a shared genetic background between smoking, BMI and BP, as suggested previously [13], [24].

Replication of published results on potential pleiotropic effects of the *CHRNA5-CHRNA3-CHRNB4* gene cluster on BMI and BP in large human samples is required. It is also of interest whether the effect is purely pleiotropic or if smoking acts as a mediator or a moderator of the associations between the gene cluster and BMI/BP, as suggested by some studies [13], [25]. Evidence for associations between variation in this gene cluster and BMI/BP will also be of interest from the point of view of identifying additional, novel susceptibility loci for BMI or BP, since identification of genes involved especially in the regulation of BP has proven challenging compared to other complex traits [29].

In the present study we have tested the effects of genetic variation within *CHRNA5-CHRNA3-CHRNB4* on BMI and BP and whether these effects are modified by smoking status. Our study adds further evidence to the previously reported association between variation at 15q25 and BMI in smokers. Moreover, ours is the first study, conducted in a large population-based birth cohort of over 5400 individuals, to suggest an interactive effect on BP as well.

## Methods

### Study population

**Figure 1:**
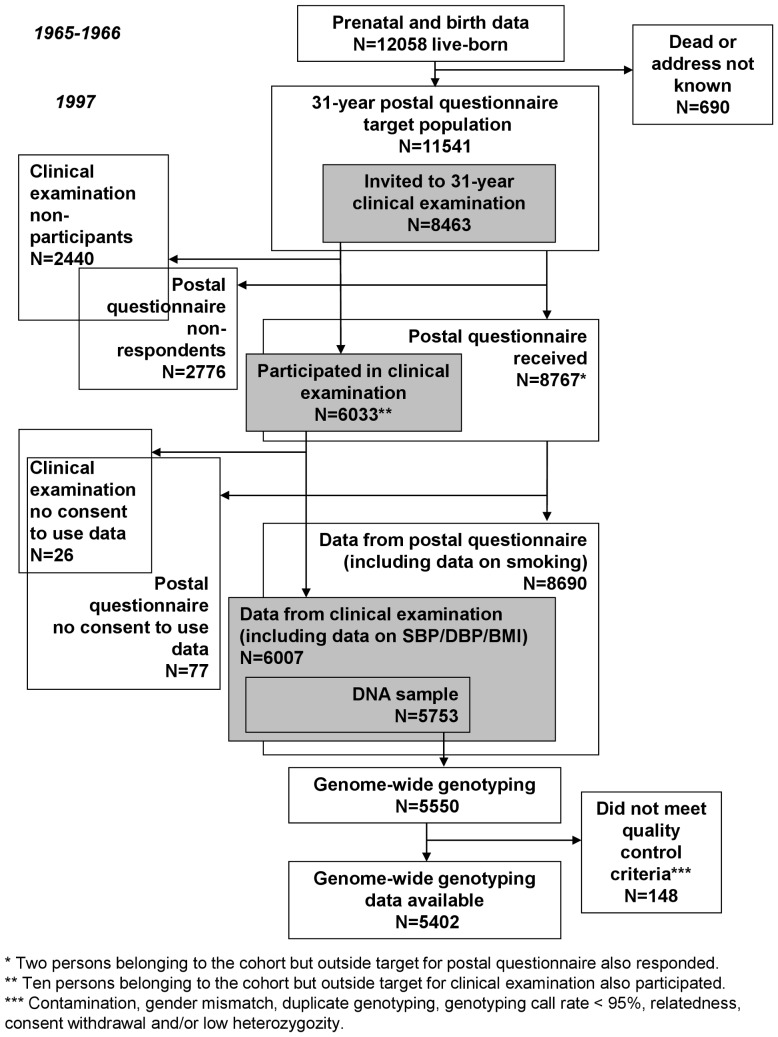
Flow chart of the NFBC1966 data collections.

The study sample consisted of members from the Northern Finland Birth Cohort 1966 (NFBC1966). The cohort was initiated in 1965 by inviting all pregnant mothers from the two northernmost provinces of Finland with their expected date of delivery occurring in 1966 to participate. The cohort covered over 96% of all births in the target area in 1966 (N = 12055 mothers with 12058 live-born children). [30] The children born to the cohort have been followed-up since the 24^th^ week of gestation until adulthood, with the latest data collection being conducted at the age of 31 years. At that point, questionnaires on health, lifestyle and occupation were mailed to all cohort members alive with known address (N = 11541). The questionnaire was returned by 75% (N = 8767) of the individuals. Those living in the original target area or in the capital (Helsinki) area (N = 8463) were also invited to a clinical examination, to which 71% (N = 6033) participated. [31] Blood samples were drawn, and DNA was extracted successfully for 5753 participants. The present sample includes those with genome-wide genotype data with an informed written consent to use their data (N = 5402) (Figure 1). The Ethics Committees of the University of Oulu and Northern Ostrobothnia Hospital District approved the study.

### Phenotypic and genetic data

Phenotypic data were obtained from the questionnaire and the clinical examination at 31 years. Smoking was assessed from questions measuring current smoking behavior (during the past year) and smoking quantity. Based on those we formed a three-category variable as follows: 1) non-smokers (including never and former smokers), 2) light smokers (1–10 cigarettes/day), and 3) heavy smokers (>10 cigarettes/day). BMI (kg/m^2^) was calculated from measured height and weight. SBP and DBP were measured twice with a mercury sphygmomanometer with a cuff size of 14 cm×40 cm in a sitting position from the right arm after 15 minutes of rest by trained nurses using a standardized procedure and ongoing quality control [32]. We used the average of the two measurements. For those who on the same 31-year questionnaire reported as being on medication for hypertension (N = 95), we added 15 mmHg to the recorded SBP values and 10 mmHg to the recorded DBP values in accordance with earlier genome-wide association studies on BP [33].

Genome-wide genotyping was performed with Illumina HumanCNV370DUO Analysis Beadchip platform at the Broad Institute, USA. Detailed genotyping and sample quality control of the first set of data (N = 4936 individuals) have been reported previously. [34] Afterwards more samples were genotyped with the same method as before (new data including also the previously genotyped set of individuals, N = 5550). In the quality control phase, 148 samples were excluded because of contamination, gender mismatch between genotypic and phenotypic gender, duplicate genotyping, genotyping call rate <95%, relatedness (IBS pairwise sharing >0.20), consent withdrawal and low heterozygozity. After quality control 5402 individuals remained available for the analysis.

Principal components (PC) to control for potential population stratification in the subsequent association analyses were calculated from the 22 directly genotyped autosomes such that all SNPs had MAF >1%, *P*-value for Hardy-Weinberg Equilibrium >0.005, call rate >99.5% and no two SNPs were in LD of r^2^>0.2 with any other selected SNP. The data were further thinned such that 1 in 15 SNPs was selected for principal component analysis. Eigenvalue decomposition was performed using the eigen function in the statistical software R [35]. It has previously been shown that the principal components calculated from these data correspond well to the geographical background of the cohort members [34].

### Statistical Analysis

For genetic data, we selected all available directly genotyped SNPs from the genome-wide data to cover the *CHRNA5-CHRNA3-CHRNB4* gene cluster and its surrounding area as in our previous publication [15]. We included only common SNPs (MAF>5%) according to the established practice in many genome-wide association studies based on concerns about low statistical power to detect associations of less common variants. These selections resulted in 18 eligible SNPs for the analyses (Table 1). The association analyses between the 18 SNPs and BMI/BP were run in Plink v1.06 [36], [37] assuming an additive effect for the genotype and adjusting the analyses for gender, BMI (for the analysis with SBP/DBP as the outcome) and three first PCs to control for potential population stratification. The distributions of the residuals of BMI, SBP and DBP did not seriously deviate from normality (data not shown); thus, the outcome variables were analyzed on their original scales assuming they follow Gaussian distributions.

**Table 1:** Selected SNPs and their characteristics.

Chr	rs number	Nearest gene	Position	Location	Effect/other allele^a^	MAF^b^
15	rs8034191	*LOC123688*	76593078	Intron	**G**/A	0.33
15	rs3885951	*LOC123688*	76612972	Flanking 3′UTR	**G**/A	0.06
15	rs2036534	*LOC123688*	76614003	Flanking 3′UTR	A/**G**	0.28
15	rs6495306	*CHRNA5*	76652948	Intron	A/**G**	0.38
15	rs680244	*CHRNA5*	76658343	Intron	G/**A**	0.38
15	rs621849	*CHRNA5*	76659916	Intron	A/**G**	0.38
15	rs1051730	*CHRNA3*	76681394	Coding	**A**/G	0.32
15	rs6495309	*CHRNB4*	76702300	Flanking 3′UTR	G/**A**	0.27
15	rs1948	*CHRNB4*	76704454	3′UTR	G/**A**	0.34
15	rs950776	*CHRNB4*	76713073	Intron	A/**G**	0.33
15	rs12594247	*CHRNB4*	76733688	Flanking 5′UTR	**A**/G	0.21
15	rs12900519	*CHRNB4*	76736182	Flanking 5′UTR	A/**G**	0.14
15	rs1996371	*CHRNB4*	76743861	Flanking 5′UTR	**G**/A	0.35
15	rs6495314	*CHRNB4*	76747584	Flanking 5′UTR	**C**/A	0.35
15	rs8032156	*CHRNB4*	76751553	Flanking 5′UTR	G/**A**	0.30
15	rs8038920	*CHRNB4*	76761600	Flanking 5′UTR	G/**A**	0.27
15	rs4887077	*CHRNB4*	76765419	Flanking 5′UTR	**A**/G	0.33
15	rs11638372	*CHRNB4*	76770614	Flanking 5′UTR	**A**/G	0.33

a Effect allele is the smoking-increasing allele (as in [15]). Minor allele is in bold.

b Minor allele frequency.

We performed the analyses separately in non-smokers and smokers, and in all subjects together, including a term for gene-environment (SNP×smoking) interaction, too. To account for multiple testing, we performed parametric bootstrap for gene-environment interactions [38] and applied the maxT multiple testing procedure, in which we compared each observed test statistic against the maximum of all bootstrapped test statistics within each simulation. This controls for the family-wise error rate, but also takes adequately into account the dependence structure between test statistics, i.e. the correlation structure between SNPs is preserved. Thus, a less stringent adjustment for multiple testing is obtained in comparison to the conventionally applied Bonferroni correction, which assumes all tests are mutually independent. The number of simulations was 10000, and within each simulation we took N = 5402 resamples, i.e. the original sample size. Simulations were performed in R [35]. The results are presented as estimated regression coefficients with their 95% confidence intervals (CI), and we report both the unadjusted and adjusted *P*-values for the interaction terms.

Additionally, we performed a series of supplemental analyses. We examined dose-response effects by separating smokers into light and heavy smokers, and differences between never, former and current smokers were tested by separating the non-smoker group into never and former smokers. Also, we tested for possible gender×SNP interactions. The analyses were performed in Plink v1.06 [36], [37] and were adjusted for the same covariates as before. Parametric bootstrap was performed as previously, and both unadjusted and adjusted *P*-values are reported.

Finally, we performed haplotype analyses to increase power and to refine the location of the susceptibility locus, because multiple SNPs may “tag” an untyped variant more effectively than a single typed variant [39]. We visualized the haplotype structure of the region with Haploview v3 [40] and identified two haplotype blocks by visual inspection. Possible haplotypes in the two blocks for each individual were phased with Plink v1.06 [36], [37] (using the Expectation-Maximization algorithm-based phasing), and the haplotype frequencies and consequent association analyses were estimated using the same software. We performed sliding window analyses [41] within the blocks, i.e. tested for associations between haplotypes of differing lengths and the outcome of interest, and we used only haplotypes with estimated frequency >1% in the sample. These analyses were run assuming an additive effect for the haplotype, i.e. 0, 1 or 2 copies of the haplotype, and adjusting for the same covariates as before. The analyses were run separately in non-smokers and smokers, and no interaction analyses were performed. To account for multiple testing, we performed maxT permutation of residuals [42], [43] in Plink with 10000 permutations. We report both the unadjusted and adjusted *P*-values.

## Results

### Distributions of the study variables

Characteristics of the study sample are presented in Table 2. Among participants with both genotypic and phenotypic data available, 58% were non-smokers and 42% smokers, of whom about half were light and half heavy smokers. Men were more likely to be heavy smokers than women (31% vs. 12%), and they had on average higher SBP and DBP compared to women. There were no obvious differences in the distributions of BMI in different smoking groups in either gender. We also checked the distributions of BMI in prolonged smokers [15] by combining data on smokers from both the 14-year and the 31-year postal questionnaires, but the average BMI of persistent smokers did not differ from that of other smokers (data not shown). SBP and DBP were slightly lower in smokers compared to non-smokers in both men and women.

**Table 2:** Sample characteristics of the NFBC1966 study sample.

		BMI	SBP	DBP
	N (%)	Mean (SD)	Mean (SD)	Mean (SD)
**Males (N = 2592)**				
Non-smokers	1219 (51.3)	25.2 (3.5)	131.1 (13.2)	81.1 (11.8)
1–10 cigarettes/day	431 (18.1)	25.0 (3.6)	129.9 (12.5)	80.2 (11.2)
>10 cigarettes/day	726 (30.6)	25.3 (3.8)	129.8 (13.3)	79.7 (11.5)
**Females (N = 2810)**				
Non-smokers	1679 (64.1)	24.0 (4.5)	120.8 (12.7)	75.4 (10.8)
1–10 cigarettes/day	626 (23.9)	24.5 (5.0)	119.3 (12.3)	73.6 (11.2)
>10 cigarettes/day	315 (12.0)	24.5 (5.6)	118.6 (13.2)	73.4 (11.6)

### Analyses on non-smokers and smokers

**Figure 2:**
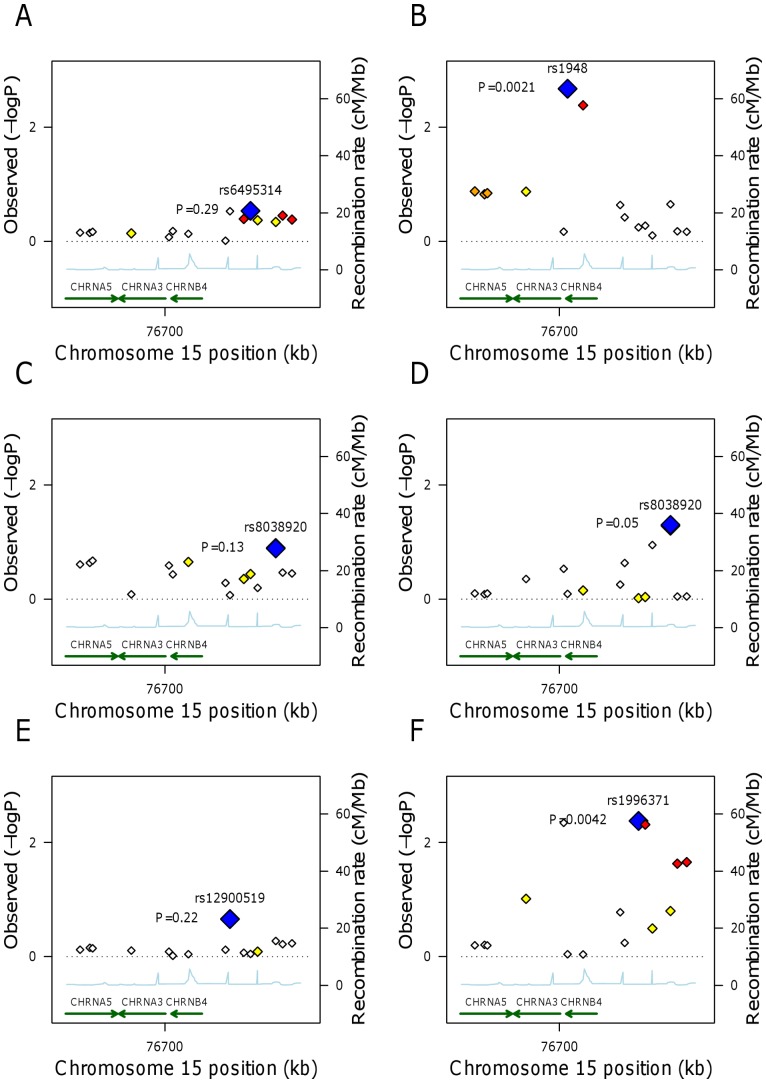
Associations between the *CHRNA5-CHRNA3-CHRNB4* gene cluster on the three outcomes of interest. (A) Systolic blood pressure, non-smokers. (B) Systolic blood pressure, smokers. (C) Diastolic blood pressure, non-smokers. (D) Diastolic blood pressure, smokers. (E) Body mass index, non-smokers. (F) Body mass index, smokers. Blue diamond indicates the most statistically significantly associated SNP, and other SNPs in the region are presented by diamonds with coloring from white to red corresponding to r^2^ values from 0 to 1. The SNP position refers to the NCBI build 35. Estimated recombination rates are from HapMap, and gene annotations from University of California at Santa Cruz genome browser with build 35 coordinates.

Table 3 shows the estimated associations between the 18 variants in the gene cluster and SBP in non-smokers and smokers. Similar results for DBP and BMI are presented in Tables 4 and 5. The results are further illustrated in Figure 2, from which both the correlation structure between the SNPs and the estimated recombination rates can also be seen. There was moderate evidence for associations between two SNPs (rs1948 and rs950776) and SBP, and six SNPs (rs2036534, rs6495309, rs1996371, rs6495314, rs4887077 and rs11638372) and BMI; evidence for associations with DBP was weaker. All the observed associations were in such a direction that the smoking-increasing allele (as reported in [15]) predicted lowered SBP, DBP or BMI, and the observed associations were generally stronger among smokers. Each additional copy of the rs1948 G-allele and rs950776 A-allele were associated with a reduction of on average −1.21 (−2.01, −0.40) mmHg in SBP in smokers. The variants associated with BMI had an average effect size of −0.38 (−0.68, −0.08) kg/m^2^ per an additional copy of the risk allele in smokers. The results from interaction models appeared to provide some support of the interactive effects observed in the stratified analyses; however, after adjustment for multiple testing the evidence for interactions was quite weak (see unadjusted and adjusted *P*-values for interaction in Tables 3, 4 and 5).

**Table 3:** Estimated associations between variants in the 15q25 region and SBP by smoking status (non-smokers, smokers) in the NFBC1966.

		Non-smokers	Smokers		
		(N = 2758–2771)	(N = 2028–2042)		
rs number	Effect/other allele^a^	beta (95% CI)^b^	beta (95% CI)^b^	*P*-value for interaction^c^	Adjusted *P*-value for interaction^d^
rs8034191	**G**/A	−0.07 (−0.76, 0.62)	−0.21 (−0.99, 0.56)	0.71	1.00
rs3885951	**G**/A	0.37 (−1.01, 1.74)	−0.43 (−1.93, 1.07)	0.40	1.00
rs2036534	A/**G**	−0.03 (−0.75, 0.69)	0.43 (−0.40, 1.25)	0.45	1.00
rs6495306	A/**G**	−0.13 (−0.80, 0.54)	−0.58 (−1.34, 0.18)	0.36	0.99
rs680244	G/**A**	−0.13 (−0.80, 0.54)	−0.56 (−1.32, 0.20)	0.38	0.99
rs621849	A/**G**	−0.14 (−0.81, 0.53)	−0.57 (−1.33, 0.19)	0.38	0.99
rs1051730	**A**/G	−0.13 (−0.83, 0.57)	−0.60 (−1.38, 0.18)	0.31	0.99
rs6495309	G/**A**	0.08 (−0.65, 0.80)	0.18 (−0.66, 1.02)	0.90	1.00
rs1948	G/**A**	−0.15 (−0.82, 0.52)	−1.24 (−2.03, −0.45)	0.04	0.49
rs950776	A/**G**	−0.12 (−0.80, 0.57)	−1.17 (−1.97, −0.37)	0.04	0.55
rs12594247	**A**/G	0.02 (−0.80, 0.83)	−0.56 (−1.47, 0.35)	0.34	0.99
rs12900519	A/**G**	−0.49 (−1.40, 0.42)	−0.49 (−1.57, 0.59)	0.98	1.00
rs1996371	**G**/A	−0.30 (−0.99, 0.40)	−0.23 (−1.00, 0.55)	0.98	1.00
rs6495314	**C**/A	−0.37 (−1.07, 0.32)	−0.25 (−1.02, 0.52)	0.90	1.00
rs8032156	G/**A**	0.29 (−0.42, 1.00)	0.11 (−0.69, 0.91)	0.75	1.00
rs8038920	G/**A**	−0.28 (−1.01, 0.45)	−0.52 (−1.36, 0.32)	0.57	1.00
rs4887077	**A**/G	−0.33 (−1.04, 0.37)	−0.17 (−0.96, 0.61)	0.86	1.00
rs11638372	**A**/G	−0.29 (−1.00, 0.41)	−0.17 (−0.95, 0.62)	0.91	1.00

a Effect allele is the smoking-increasing allele (as in [15]). Minor allele is in bold.

b Linear regression model including SNP, gender, BMI at 31 years, three first PCs.

c Interaction model including SNP, gender, BMI at 31 years, smoking (no,yes), three first PCs, SNP*smoking.

d Adjustment for multiple testing by MaxT bootstrap test for gene-environment interaction.

**Table 4 pone-0046557-t004:** Estimated associations between variants in the 15q25 region and DBP by smoking status (non-smokers, smokers) in the NFBC1966.

		Non-smokers	Smokers		
		(N = 2755–2768)	(N = 2025–2039)		
rs number	Effect/other allele^a^	beta (95% CI)^b^	beta (95% CI)^b^	*P*-value for interaction^c^	Adjusted *P*-value for interaction^d^
rs8034191	**G**/A	0.08 (−0.52, 0.68)	−0.14 (−0.83, 0.54)	0.58	1.00
rs3885951	**G**/A	0.12 (−1.07, 1.31)	0.14 (−1.18, 1.46)	0.98	1.00
rs2036534	A/**G**	0.43 (−0.20, 1.06)	−0.12 (−0.85, 0.60)	0.24	0.99
rs6495306	A/**G**	−0.34 (−0.92, 0.24)	−0.09 (−0.76, 0.58)	0.60	1.00
rs680244	G/**A**	−0.35 (−0.93, 0.23)	−0.08 (−0.74, 0.59)	0.57	1.00
rs621849	A/**G**	−0.37 (−0.95, 0.21)	−0.09 (−0.76, 0.58)	0.57	1.00
rs1051730	**A**/G	0.07 (−0.54, 0.67)	−0.27 (−0.96, 0.42)	0.41	0.99
rs6495309	G/**A**	0.36 (−0.27, 1.00)	−0.39 (−1.13, 0.34)	0.12	0.89
rs1948	G/**A**	−0.27 (−0.85, 0.32)	−0.09 (−0.78, 0.61)	0.74	1.00
rs950776	A/**G**	−0.37 (−0.96, 0.23)	−0.14 (−0.84, 0.57)	0.66	1.00
rs12594247	**A**/G	−0.23 (−0.93, 0.47)	−0.24 (−1.04, 0.56)	0.98	1.00
rs12900519	A/**G**	−0.08 (−0.87, 0.71)	0.58 (−0.37, 1.53)	0.35	0.99
rs1996371	**G**/A	−0.24 (−0.84, 0.37)	−0.02 (−0.70, 0.66)	0.72	1.00
rs6495314	**C**/A	−0.28 (−0.88, 0.32)	−0.04 (−0.72, 0.65)	0.68	1.00
rs8032156	G/**A**	−0.15 (−0.76, 0.47)	−0.57 (−1.27, 0.13)	0.43	1.00
rs8038920	G/**A**	−0.50 (−1.13, 0.14)	−0.73 (−1.47, 0.01)	0.62	1.00
rs4887077	**A**/G	−0.29 (−0.90, 0.31)	−0.04 (−0.73, 0.65)	0.67	1.00
rs11638372	**A**/G	−0.29 (−0.90, 0.32)	−0.05 (−0.74, 0.65)	0.69	1.00

a Effect allele is the smoking-increasing allele (as in [15]). Minor allele is in bold.

b Linear regression model including SNP, gender, BMI at 31 years, three first PCs.

c Interaction model including SNP, gender, BMI at 31 years, smoking (no,yes), three first PCs, SNP*smoking.

d Adjustment for multiple testing by MaxT bootstrap test for gene-environment interaction.

**Table 5:** Estimated associations between variants in the 15q25 region and BMI by smoking status (non-smokers, smokers) in the NFBC1966.

		Non-smokers	Smokers		
		(N = 2758–2771)	(N = 2028–2042)		
rs number	Effect/other allele^a^	beta (95% CI)^b^	beta (95% CI)^b^	*P*-value for interaction^c^	Adjusted *P*-value for interaction^d^
rs8034191	**G**/A	0.02 (−0.21, 0.26)	−0.26 (−0.54, 0.03)	0.14	0.94
rs3885951	**G**/A	−0.09 (−0.55, 0.37)	0.09 (−0.46, 0.64)	0.57	1.00
rs2036534	A/**G**	0.00 (−0.24, 0.24)	−0.35 (−0.65, −0.05)	0.08	0.79
rs6495306	A/**G**	0.04 (−0.19, 0.26)	0.07 (−0.21, 0.35)	0.81	1.00
rs680244	G/**A**	0.04 (−0.18, 0.27)	0.07 (−0.21, 0.35)	0.84	1.00
rs621849	A/**G**	0.04 (−0.18, 0.26)	0.07 (−0.21, 0.35)	0.84	1.00
rs1051730	**A**/G	0.03 (−0.20, 0.27)	−0.24 (−0.53, 0.04)	0.17	0.96
rs6495309	G/**A**	−0.03 (−0.27, 0.21)	−0.44 (−0.75, −0.14)	0.03	0.46
rs1948	G/**A**	0.01 (−0.22, 0.23)	−0.02 (−0.31, 0.27)	0.97	1.00
rs950776	A/**G**	−0.01 (−0.24, 0.21)	0.02 (−0.28, 0.31)	0.83	1.00
rs12594247	**A**/G	0.04 (−0.23, 0.31)	0.24 (−0.10, 0.57)	0.38	0.99
rs12900519	A/**G**	−0.19 (−0.49, 0.11)	0.11 (−0.28, 0.51)	0.17	0.96
rs1996371	**G**/A	−0.02 (−0.25, 0.21)	−0.41 (−0.70, −0.13)	0.05	0.57
rs6495314	**C**/A	−0.02 (−0.25, 0.22)	−0.41 (−0.69, −0.12)	0.05	0.57
rs8032156	G/**A**	0.03 (−0.21, 0.27)	0.15 (−0.15, 0.45)	0.64	1.00
rs8038920	G/**A**	0.08 (−0.17, 0.32)	−0.22 (−0.53, 0.09)	0.11	0.89
rs4887077	**A**/G	−0.06 (−0.30, 0.17)	−0.33 (−0.62, −0.05)	0.19	0.97
rs11638372	**A**/G	−0.07 (−0.30, 0.17)	−0.34 (−0.62, −0.05)	0.19	0.97

a Effect allele is the smoking-increasing allele (as in [15]). Minor allele is in bold.

b Linear regression model including SNP, gender, three first PCs.

c Interaction model including SNP, gender, smoking (no, yes), three first PCs, SNP*smoking.

d Adjustment for multiple testing by MaxT bootstrap test for gene-environment interaction.

Because the gene cluster has previously been associated with smoking quantity, we also considered the possibility that the associations found could be due to residual confounding by smoking quantity. Therefore we reran the analyses on smokers adjusted by a continuous measure of smoking quantity (cigarettes/day), but the results remained practically unchanged (data not shown).

### Analyses on light and heavy smokers

For SBP, the observed effects were slightly stronger in heavy smokers than in light smokers; e.g. for rs1948 the G-allele was associated with 0.97 mmHg lower SBP (95% CI −2.20, 0.13) in light smokers whereas in heavy smokers the corresponding figures were −1.50 (−2.64, −0.37) (Table S1). For DBP, some suggestive associations that were previously seen in smokers became more evident in heavy smokers; e.g. for rs8038920 each additional copy of the G-allele predicted 0.73 mmHg lower DBP (−1.47, 0.01) in smokers while in heavy smokers the predicted reduction was −1.40 (−2.44, −0.35) mmHg/one copy of the G-allele (Table S2). In the analysis of BMI, for some of the SNPs (rs8034191, rs1051730 and rs6495309) the effects seemed to be strongest in light smokers (Table S3). The formal assessments of interaction did not give adequate support to these findings, though, especially after adjustment for multiple testing.

### Analyses on never, former and current smokers

For the SNPs on which we had observed evidence for associations in current smokers (rs1948 and rs950776 with SBP; rs2036534, rs6495309, rs1996371, rs6495314, rs4887077 and rs11638372 with BMI), the associations were attenuated in former smokers (Tables S4,S5,S6). For one of the SNPs (rs12900519), however, the effect on SBP seemed to be stronger in former (−1.58 (−3.04, −0.13) mmHg/each additional copy of A-allele) than in current smokers (−0.49 (−1.57, 0.59) mmHg/each additional copy of the A-allele) (Table S4).

### Gender interactions

Since the distributions of SBP, DBP and BMI differed by gender (Table 2), we studied if there is heterogeneity by gender also on the associations between the 18 SNPs and the three outcomes of interest. For the two SNPs (rs1948 and rs950776) on which we found evidence for association with SBP when the genders were analyzed together (Table 3), the effect seemed superficially to be stronger among females (Table S7). Also for an additional SNP (rs12900519) we observed a stronger effect in females. For DBP, six SNPs predicted lower DBP in females but not in males, and one SNP (rs6495309) was associated with opposing effects among males and females (Table S8). Instead for BMI, many of the associations observed when the genders were analyzed together (Table 5) seemed to be driven by stronger associations in males (Table S9). However, for all of these findings by gender, the support from interaction analyses was weak.

### Multimarker analyses

**Figure 3:**
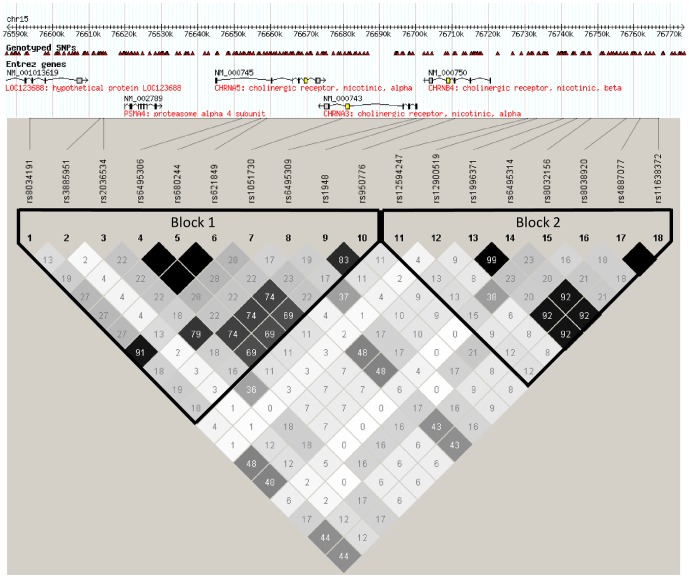
The linkage disequilibrium (LD) structure of the chromosome 15q25 region. Identified haplotype blocks 1 and 2 are indicated with borders. R^2^ values are presented using grayscale coloring from white to black corresponding to r^2^ values from 0 to 1.

The linkage disequilibrium (LD) plot of the region is displayed in Figure 3. The estimated haplotype frequencies and regression coefficients with their 95% CIs are available in Tables S10 (results for haplotypes in block1) and S11 (results for haplotypes in block2). The results are presented only for those haplotypes with an estimated frequency >1% and with an unadjusted *P*-value<0.05. Such results were observed only in smokers. In the single marker analysis we observed evidence for associations for SNPs from the first block and SBP, SNPs from the second block and DBP and SNPs from both blocks and BMI (Tables 3–5). The haplotype analyses were in line with this, since there was no strong evidence for haplotypes from block 2 to be associated with SBP, nor for haplotypes from block 1 with DBP, whereas there was evidence for associations between haplotypes from both blocks and BMI (Tables S10, S11).

In more detail, for SBP, and after adjustment for multiple testing, there was moderate evidence for a SBP-lowering haplotype consisting of the G-allele of rs1948 and A-allele of rs950776; the same alleles of the two SNPs that were associated with lower SBP in the single marker analyses. Instead, SBP-increasing haplotypes included the A and G alleles of the above-mentioned SNPs, respectively. The pattern for the alleles of the other SNPs included in SBP-associated haplotypes was not as clear-cut as it was for rs1948 and rs950776; the same alleles in different haplotypes could be associated either with increased or decreased SBP (Table S10).

The estimated effect sizes on SBP were bigger for many SBP-increasing haplotypes than for any of the SBP-lowering haplotypes or single SNPs in the single marker analysis (Table S10). An increase of about 6 mmHg in SBP per each additional copy of the haplotype was observed for six haplotypes of varying lengths. All of these haplotypes were estimated to be rare in the population with an allele frequency of about 1%.

Regarding BMI and the haplotypes from the first block, there was no strong evidence for associations after adjustment for multiple testing (Table S10). In the second block (Table S11), we observed some evidence for associations of the whole block-spanning haplotype GAGCGGAA or haplotypes of differing lengths but with the same alleles as in the long haplotype and lower BMI, except that in some cases the two last alleles were AA instead of GG. Again, bigger effect sizes were observed in multimarker analyses than in single marker analyses, but these effects were arising from haplotypes with low estimated population frequencies (Table S11).

## Discussion

### Main findings

The present study suggests that in addition to the previously presented hypotheses for the association between increased smoking and lower BP, there might be shared a genetic architecture behind as well. In the single marker analyses, weak evidence was found for associations between two polymorphisms from the smoking-associated *CHRNA5-CHRNA3-CHRNB4* gene cluster and lower BP. We found weak evidence for associations between smoking-increasing alleles and lower BMI as well, although the distributions of BMI did not vary across different smoking strata. If same alleles predisposed to smoking and lower BMI, we would have expected to observe on average lower BMI in smokers, which was not the case in our data. However, we believe that the association between smoking and BMI is highly confounded by socio-behavioral factors, for which we did not control when comparing the distributions of BMI across different smoking strata, because addressing the associations between smoking and BMI/BP in detail was beyond the scope of the present study.

Different SNPs were associated with BP and BMI, and even with SBP and DBP. Mainly markers from *CHRNB4* were associated with SBP, pointing towards β4 subunits being mostly responsible for these effects, whereas markers from *CHRNA5* and *CHRNB4* were associated with BMI, emphasizing the role of α5 and β4 subunits in affecting BMI. However, further studies are needed to locate the real causal variants and the receptor subunits responsible for the observed associations. For all of the six SNPs on which we found evidence for any association with BMI, the r^2^ with rs1948, the most strongly associated SNP with SBP, was <0.20 indicating that the SNPs associated with BMI and SBP seem to be nearly independent of each other. The haplotype association analyses also supported this finding since mainly haplotypes from block 1 were associated with SBP, haplotypes from block 2 with DBP, and haplotypes from both blocks with BMI. The haplotype analyses also provided further support for the two SNPs found to be associated with SBP in the single marker analyses; the haplotype GA consisting of rs1948 and rs950776 predicted lower SBP even after correction for multiple testing. For BMI, the findings from the haplotype analyses were less consistent with the findings from the single marker analyses.

The results from the additional, rather explorative analyses suggested that the effects of the SNPs on SBP would be stronger in heavy smokers than in light smokers. The associations between the SNPs and SBP/BMI were attenuated in former smokers, suggesting that the associations would weaken over time after cessation. In addition, there might be heterogeneity by gender in the associations. However, additional stratifications reduced the sample sizes resulting in very wide confidence intervals, and moreover, the interaction analyses provided no adequate support for these findings. Studies with larger sample sizes are needed to find further support for these suggestive results.

We also tried to address the fact the possibility that the associations seen are due to residual confounding by smoking quantity. By adjusting the analyses in smokers by a continuous measure of cigarettes/day did not change the results obtained previously, suggesting that the associations seen are not due to residual confounding. However, in all of the analyses the measure of smoking was self-reported, possibly leading to underestimation of the true effect. For example, the availability and the use of cotinine levels might control better for the actual quantity of smoked cigarettes.

### Comparison with previous studies on the gene cluster

Of the SNPs that we found evidence for an association with BP, rs1948 in *CHRNB4* has previously been associated with heavy smoking in adulthood [15], nicotine dependence [44] and with early age of initiation of tobacco and alcohol use [45]. Another SNP for which we observed evidence for an association with lower BMI, rs1996371 and especially its allele G, has previously been associated with heavy smoking and nicotine dependence [7], [12], [15]. However, none of the SNPs that appeared to be associated with SBP/BMI in the present study have previously reached genome-wide significance and thus been identified as potential susceptibility loci for BMI or BP according to the NHGRI catalog of published genome-wide association studies [46] or based on our literature search.

Only two SNPs from the *CHRNA5-CHRNA3-CHRNB4* gene cluster have previously been reported to be associated with BMI or SBP. SNP rs1051730 in *CHRNA3* reported to be associated with BMI [25] and which is also the strongest signal previously associated with smoking quantity, was not very strongly associated either with BP or BMI in the present study. However, the effects were in the direction that the smoking-associated A-allele was predictive of decreased SBP and BMI in smokers, which is in line with the previous publication reporting lower BMI in smokers carrying the A-allele [25]. The other SNP, rs3743075 in *CHRNA3*, reported to be associated with lower SBP in twins [24] was not included in our directly genotyped data.

### Possible mechanisms behind the associations

Co-morbidity between addictive behaviors of smoking, alcohol use and eating disorders has been reported [47], [48] suggesting a common underlying neurobiological mechanism. In this respect, it would not be surprising if same variants were associated with both smoking and BMI. In addition, nAChRs are plausible candidates for this common mechanism because they have been associated with different types of addictive behaviors: nicotine dependence [1], [2], [4], [6], [7], [9], [13], alcohol use [49] and overweight and obesity [50]. Further, evidence has been documented on an association between the *CHRNA5-CHRNA3-CHRNB4* gene cluster and activity of the dorsal anterior cingulate-ventral striatum/extended amygdala circuit, which is an important circuit in modulating reward to drugs such as alcohol or smoking as well as food [51]. The associations of variation within genes encoding for nAChR subunits and BMI in the present study add evidence to this common mechanism hypothesis but future research is needed to better understand the biology behind.

The mechanisms by which the nAChRs regulate BP are also unclear. The genetic variants were still found to be associated with SBP even after adjustment for BMI suggesting a direct effect (i.e. not mediated via BMI) of these loci on SBP. In line with this idea, the genetic variants associated with SBP were different from those associated with BMI in our sample and they seemed to be nearly independent based on the low r^2^ between the variants associated with SBP and BMI. This suggests that the *CHRNA5-CHRNA3-CHRNB4* gene cluster regulates SBP through biological mechanisms that partly differ from those regulating BMI. One plausible mechanism is that the nicotinic receptors modulate SBP by influencing the autonomic function. In this regard, Rana *et al.* showed that polymorphisms in the region were associated with catestatin, a peptide regulating cardiac function and blood pressure. The catestatin peptide fragment of the endogenous catecholamine secretory vesicle protein chromogranin A (CHGA) acts as an antagonist of the nicotinic cationic channels and regulates catecholamine release. This led the authors to suggest that nAChRs may regulate BP via mediating release of catestatin. Indeed, the same variants associated with catestatin were also associated with SBP, and cathecolamine levels. [24] We were not able to test this or other biologically plausible pathways due to the lack of appropriate biological markers measured in the cohort.

In the present study the associations between variation in the gene cluster and BMI/BP appeared to be more pronounced in smokers, suggesting that smoking would be a modifier of these associations. However, after adjustment for multiple testing, there was no sufficient evidence for interactive effects. The shortage of evidence for interaction might be due to limited power of our study to detect gene-environment interactions. Previous studies have reported associations between polymorphisms in the gene cluster and BMI manifest only in smokers [13], [25] or in alcohol consumers [49]. For BP, an interaction with smoking has not been reported before. Rana *et al.* showed associations between polymorphisms in the gene cluster and SBP adjusted for smoking, but they did not test for interactive effects in their study [24]. Although it seems that smoking is a favorable factor when carrying certain alleles as they are associated with lower BP and BMI especially in smokers, we cannot consider smoking beneficial in general because it is still an important risk factor for CVD. The identification of variants that lower BP and BMI in smokers, however, adds to the knowledge of pleiotropic effects of the variants in the gene cluster. Hopefully this could also be exploited in future development of medication by imitating the mechanisms through which nicotinic acetylcholine receptors influence BP and BMI in smokers.

### Strengths and limitations

The analyses were performed in a population-based birth cohort including individuals of Northern European ancestry with a fairly homogeneous background. The study participants were of relatively young age (31 years) at the time of BMI and BP assessment, and as BP tends to increase with age, the effects could be stronger in older cohorts. In addition, age-varying associations are also possible [52]. Otherwise, previous GWA studies including NFBC have shown that the cohort is well comparable and the results are generalizable with other populations from similar ancestral background (e.g. [53], [54]).

However, replication is still required in populations of European ancestry, because although the sample size was moderately large compared to some of the previous studies, e.g. the 370 twins in the study for the gene cluster and SBP [24], the statistical evidence was not particularly strong but suggestive and especially regarding gene-environment interactions, the power was quite limited. The identification of gene-environment interactions is challenging, as was shown by Figueiredo *et al.* [55] in their recent study in which they compared three different methods for finding gene-environment interactions in an association study setting. Thomas *et al.* [56] further discuss the issues related to the search of gene-environment interactions, highlighting well-designed studies with careful measurement and efficient analysis of both genetic and environmental factors.

The replication of our suggestive results, and addressing further gene-environment, such as gender differences, or gene-gene interactions, would therefore require substantially larger numbers of individuals. Cohorts with certain biological markers will also be valuable in the search of specific pathways leading from nAChRs to cardiovascular risk factors.

### Summary and conclusions

Our results suggest that variation in *CHRNA5-CHRNA3-CHRNB4* is associated with cardiovascular risk factors and that smoking possibly acts as a modifier of these associations. Large collaborative efforts are needed to find further evidence for the associations, to test for their existence in other ancestral populations and age groups, and to understand the mechanisms by which the nAChRs affect BMI and BP.

## Supporting Information

Table S1 **Estimated associations between variants in the 15q25 region and SBP according to smoking status (non-smokers, light and heavy smokers) in the NFBC1966.**
(PDF)

Table S2 **Estimated associations between variants in the 15q25 region and DBP according to smoking status (non-smokers, light and heavy smokers) in the NFBC1966.**
(PDF)

Table S3 **Estimated associations between variants in the 15q25 region and BMI according to smoking status (non-smokers, light and heavy smokers) in the NFBC1966.**
(PDF)

Table S4 **Estimated associations between variants in the 15q25 region and SBP according to smoking status (never, former and current smokers) in NFBC1966.**
(PDF)

Table S5 **Estimated associations between variants in the 15q25 region and DBP according to smoking status (never, former and current smokers) in the NFBC1966.**
(PDF)

Table S6 **Estimated associations between variants in the 15q25 region and BMI according to smoking status (never, former and current smokers) in the NFBC1966.**
(PDF)

Table S7 **Estimated associations between variants in the 15q25 region and SBP according to gender in the NFBC1966.**
(PDF)

Table S8 **Estimated associations between variants in the 15q25 region and DBP according to gender in NFBC1966.**
(PDF)

Table S9 **Estimated associations between variants in the 15q25 region and BMI according to gender in the NFBC1966.**
(PDF)

Table S10 **Estimated haplotype frequencies and estimates from association analyses (Block 1) for SBP and BMI in smokers in the NFBC1966.**
(PDF)

Table S11 **Estimated haplotype frequencies and estimates from association analyses (Block 2) for DBP and BMI in smokers in the NFBC1966.**
(PDF)
