# Supplementary material for: Associations between Variation in CHRNA5-CHRNA3-CHRNB4, Body Mass Index and Blood Pressure in the Northern Finland Birth Cohort 1966
Source: PLoS One. 2012 Sep 27;7(9):e46557. doi: 10.1371/journal.pone.0046557 (PMC3459914; doi:10.1371/journal.pone.0046557)
Supplement: Table S1 — Estimated associations between variants in the 15q25 region and SBP according to smoking status (non-smokers, light and heavy smokers) in the NFBC1966. (PDF) [file pone.0046557.s001.pdf]

Table S1. Estimated associations between variants in the 15q25 region and SBP according to smoking status (non-smokers, light and heavy smokers) in the NFBC1966.

		Non-smokers (N=2758-2771)	Light smokers (N=1011-1021)	Heavy smokers (N=1017-1021)				
**rs number**	**Effect/ other allele** [a]	**beta (95% CI)** [b]	**beta (95% CI)** [b]	**beta (95% CI)** [b]	***P*-value for interaction (A)** [c]	***P*-value for interaction (B)** [c]	**Adjusted *P*-value for interaction (A)** [d]	**Adjusted *P*-value for interaction (B)** [d]
rs8034191	**G**/A	-0.07 (-0.76, 0.62)	0.11 (-0.99, 1.22)	-0.51 (-1.62, 0.60)	0.82	0.44	1.00	0.99
rs3885951	**G**/A	0.37 (-1.01, 1.74)	-0.54 (-2.69, 1.60)	-0.44 (-2.55, 1.68)	0.47	0.53	1.00	1.00
rs2036534	A/**G**	-0.03 (-0.75, 0.69)	0.68 (-0.43, 1.80)	0.17 (-1.06, 1.41)	0.29	0.88	0.99	1.00
rs6495306	A/**G**	-0.13 (-0.80, 0.54)	-0.46 (-1.52, 0.59)	-0.71 (-1.81, 0.38)	0.56	0.39	1.00	0.99
rs680244	G/**A**	-0.13 (-0.80, 0.54)	-0.47 (-1.52, 0.59)	-0.66 (-1.76, 0.43)	0.55	0.43	1.00	0.99
rs621849	A/**G**	-0.14 (-0.81, 0.53)	-0.49 (-1.54, 0.57)	-0.67 (-1.76, 0.43)	0.54	0.44	1.00	0.99
rs1051730	**A**/G	-0.13 (-0.83, 0.57)	-0.23 (-1.34, 0.89)	-0.96 (-2.07, 0.15)	0.83	0.18	1.00	0.97
rs6495309	G/**A**	0.08 (-0.65, 0.80)	0.37 (-0.75, 1.50)	-0.02 (-1.27, 1.24)	0.62	0.77	1.00	1.00
rs1948	G/**A**	-0.15 (-0.82, 0.52)	-0.97 (-2.07, 0.13)	-1.50 (-2.64, -0.37)	0.20	0.04	0.98	0.55
rs950776	A/**G**	-0.12 (-0.80, 0.57)	-1.09 (-2.20, 0.01)	-1.26 (-2.42, -0.10)	0.13	0.09	0.92	0.82
rs12594247	**A**/G	0.02 (-0.80, 0.83)	-0.32 (-1.56, 0.92)	-0.85 (-2.18, 0.48)	0.58	0.32	1.00	0.99
rs12900519	A/**G**	-0.49 (-1.40, 0.42)	-1.20 (-2.71, 0.31)	0.15 (-1.41, 1.70)	0.40	0.43	0.99	0.99
rs1996371	**G**/A	-0.30 (-0.99, 0.40)	0.30 (-0.77, 1.37)	-0.70 (-1.82, 0.42)	0.38	0.41	0.99	0.99
rs6495314	**C**/A	-0.37 (-1.07, 0.32)	0.28 (-0.79, 1.36)	-0.72 (-1.84, 0.40)	0.34	0.46	0.99	1.00
rs8032156	G/**A**	0.29 (-0.42, 1.00)	0.33 (-0.78, 1.44)	-0.11 (-1.27, 1.06)	0.99	0.59	1.00	1.00
rs8038920	G/**A**	-0.28 (-1.01, 0.45)	0.45 (-0.71, 1.61)	-1.43 (-2.64, -0.23)	0.36	0.07	0.99	0.73
rs4887077	**A**/G	-0.33 (-1.04, 0.37)	0.47 (-0.62, 1.57)	-0.73 (-1.86, 0.40)	0.26	0.42	0.99	0.99
rs11638372	**A**/G	-0.29 (-1.00, 0.41)	0.47 (-0.62, 1.57)	-0.72 (-1.85, 0.41)	0.28	0.40	0.99	0.99

[a] Effect allele is the smoking-increasing allele. Minor allele is in bold.
[b] Linear regression model including SNP, gender, BMI at 31 years, three first PCs.
[c] Interaction model including SNP, gender, BMI at 31 years, smoking (no, light, heavy), three first PCs, SNP*smoking. The interaction terms are for SNP*light smoking (A) and SNP*heavy smoking (B).
[d] Adjustment for multiple testing by MaxT bootstrap test for gene-environment interaction.
