# Supplementary material for: Associations between Variation in CHRNA5-CHRNA3-CHRNB4, Body Mass Index and Blood Pressure in the Northern Finland Birth Cohort 1966
Source: PLoS One. 2012 Sep 27;7(9):e46557. doi: 10.1371/journal.pone.0046557 (PMC3459914; doi:10.1371/journal.pone.0046557)
Supplement: Table S2 — Estimated associations between variants in the 15q25 region and DBP according to smoking status (non-smokers, light and heavy smokers) in the NFBC1966. (PDF) [file pone.0046557.s002.pdf]

Table S2. Estimated associations between variants in the 15q25 region and DBP according to smoking status (non-smokers, light and heavy smokers) in the NFBC1966.

		**Non-smokers (N=2755-2768)**	**Light smokers (N=1010-1020)**	**Heavy smokers (N=1015-1019)**				
**rs number**	**Effect/ other allele[a]**	**beta (95% CI) [b]**	**beta (95% CI) [b]**	**beta (95% CI) [b]**	***P*-value for interaction (A) [c]**	***P*-value for interaction (B) [c]**	**Adjusted *P*-value for interaction (A) [d]**	**Adjusted *P*-value for interaction (B) [d]**
rs8034191	**G**/A	0.08 (-0.52, 0.68)	0.43 (-0.56, 1.42)	-0.62 (-1.58, 0.33)	0.59	0.19	1.00	0.98
rs3885951	**G**/A	0.12 (-1.07, 1.31)	0.04 (-1.88, 1.96)	0.16 (-1.67, 1.99)	0.92	0.93	1.00	1.00
rs2036534	A/**G**	0.43 (-0.2, 1.06)	0.14 (-0.86, 1.14)	-0.36 (-1.42, 0.71)	0.63	0.18	1.00	0.97
rs6495306	A/**G**	-0.34 (-0.92, 0.24)	0.15 (-0.80, 1.09)	-0.31 (-1.25, 0.64)	0.43	0.96	1.00	1.00
rs680244	G/**A**	-0.35 (-0.93, 0.23)	0.14 (-0.80, 1.09)	-0.28 (-1.23, 0.67)	0.42	0.91	1.00	1.00
rs621849	A/**G**	-0.37 (-0.95, 0.21)	0.12 (-0.83, 1.07)	-0.28 (-1.23, 0.67)	0.43	0.89	1.00	1.00
rs1051730	**A**/G	0.07 (-0.54, 0.67)	0.29 (-0.71, 1.29)	-0.73 (-1.70, 0.23)	0.79	0.14	1.00	0.93
rs6495309	G/**A**	0.36 (-0.27, 1.00)	-0.12 (-1.13, 0.89)	-0.67 (-1.75, 0.42)	0.45	0.09	1.00	0.81
rs1948	G/**A**	-0.27 (-0.85, 0.32)	0.07 (-0.92, 1.06)	-0.21 (-1.19, 0.78)	0.62	0.97	1.00	1.00
rs950776	A/**G**	-0.37 (-0.96, 0.23)	-0.10 (-1.10, 0.89)	-0.16 (-1.17, 0.84)	0.69	0.74	1.00	1.00
rs12594247	**A**/G	-0.23 (-0.93, 0.47)	-0.16 (-1.28, 0.95)	-0.31 (-1.47, 0.84)	0.99	0.95	1.00	1.00
rs12900519	A/**G**	-0.08 (-0.87, 0.71)	0.00 (-1.35, 1.36)	1.14 (-0.20, 2.48)	0.99	0.14	1.00	0.94
rs1996371	**G**/A	-0.24 (-0.84, 0.37)	0.51 (-0.45, 1.47)	-0.49 (-1.46, 0.48)	0.21	0.54	0.99	1.00
rs6495314	**C**/A	-0.28 (-0.88, 0.32)	0.49 (-0.47, 1.46)	-0.51 (-1.48, 0.46)	0.20	0.57	0.98	1.00
rs8032156	G/**A**	-0.15 (-0.76, 0.47)	-0.17 (-1.16, 0.82)	-0.96 (-1.97, 0.05)	0.95	0.22	1.00	0.99
rs8038920	G/**A**	-0.50 (-1.13, 0.14)	-0.01 (-1.06, 1.03)	-1.40 (-2.44, -0.35)	0.46	0.14	1.00	0.93
rs4887077	**A**/G	-0.29 (-0.90, 0.31)	0.52 (-0.47, 1.50)	-0.52 (-1.49, 0.46)	0.20	0.59	0.98	1.00
rs11638372	**A**/G	-0.29 (-0.90, 0.32)	0.52 (-0.47, 1.50)	-0.52 (-1.50, 0.46)	0.20	0.57	0.98	1.00

[a] Effect allele is the smoking-increasing allele. Minor allele is in bold.

[b] Linear regression model including SNP, gender, BMI at 31 years, three first PCs.

[c] Interaction model including SNP, gender, BMI at 31 years, smoking (no, light, heavy), three first PCs, SNP*smoking. The interaction terms are for SNP*light smoking (A) and SNP*heavy smoking (B).

[d] Adjustment for multiple testing by MaxT bootstrap test for gene-environment interaction.
