# Supplementary material for: Associations between Variation in CHRNA5-CHRNA3-CHRNB4, Body Mass Index and Blood Pressure in the Northern Finland Birth Cohort 1966
Source: PLoS One. 2012 Sep 27;7(9):e46557. doi: 10.1371/journal.pone.0046557 (PMC3459914; doi:10.1371/journal.pone.0046557)
Supplement: Table S3 — Estimated associations between variants in the 15q25 region and BMI according to smoking status (non-smokers, light and heavy smokers) in the NFBC1966. (PDF) [file pone.0046557.s003.pdf]

Table S3. Estimated associations between variants in the 15q25 region and BMI according to smoking status (non-smokers, light and heavy smokers) in the NFBC1966.

		Non-smokers (N=2758-2771)	Light smokers (N=1012-1022)	Heavy smokers (N=1021-1025)				
**rs number**	**Effect/ other allele**[a]	**beta (95% CI)** [b]	**beta (95% CI)** [b]	**beta (95% CI)** [b]	***P*-value for interaction (A)** [c]	***P*-value for interaction (B)** [c]	**Adjusted *P*-value for interaction (A)** [d]	**Adjusted *P*-value for interaction (B)** [d]
rs8034191	**G**/A	0.02 (-0.21, 0.26)	-0.44 (-0.86, -0.03)	-0.12 (-0.52, 0.27)	0.07	0.55	0.72	1.00
rs3885951	**G**/A	-0.09 (-0.55, 0.37)	-0.29 (-1.10, 0.51)	0.45 (-0.30, 1.21)	0.72	0.23	1.00	0.99
rs2036534	A/**G**	0.00 (-0.24, 0.24)	-0.39 (-0.81, 0.03)	-0.31 (-0.75, 0.13)	0.12	0.20	0.91	0.98
rs6495306	A/**G**	0.04 (-0.19, 0.26)	-0.03 (-0.43, 0.37)	0.13 (-0.26, 0.53)	0.87	0.59	1.00	1.00
rs680244	G/**A**	0.04 (-0.18, 0.27)	-0.03 (-0.43, 0.37)	0.14 (-0.26, 0.53)	0.85	0.61	1.00	1.00
rs621849	A/**G**	0.04 (-0.18, 0.26)	-0.03 (-0.43, 0.37)	0.13 (-0.26, 0.53)	0.84	0.61	1.00	1.00
rs1051730	**A**/G	0.03 (-0.20, 0.27)	-0.49 (-0.91, -0.07)	-0.06 (-0.46, 0.34)	0.05	0.75	0.60	1.00
rs6495309	G/**A**	-0.03 (-0.27, 0.21)	-0.49 (-0.91, -0.07)	-0.40 (-0.85, 0.04)	0.06	0.13	0.68	0.91
rs1948	G/**A**	0.01 (-0.22, 0.23)	-0.05 (-0.46, 0.37)	-0.02 (-0.43, 0.39)	0.94	1.00	1.00	1.00
rs950776	A/**G**	-0.01 (-0.24, 0.21)	-0.09 (-0.51, 0.33)	0.10 (-0.32, 0.52)	0.84	0.60	1.00	1.00
rs12594247	**A**/G	0.04 (-0.23, 0.31)	0.21 (-0.26, 0.68)	0.24 (-0.23, 0.72)	0.51	0.45	1.00	1.00
rs12900519	A/**G**	-0.19 (-0.49, 0.11)	-0.09 (-0.66, 0.48)	0.28 (-0.28, 0.84)	0.65	0.09	1.00	0.81
rs1996371	**G**/A	-0.02 (-0.25, 0.21)	-0.43 (-0.83, -0.03)	-0.42 (-0.82, -0.02)	0.11	0.10	0.88	0.86
rs6495314	**C**/A	-0.02 (-0.25, 0.22)	-0.41 (-0.81, 0.00)	-0.43 (-0.83, -0.03)	0.12	0.09	0.91	0.82
rs8032156	G/**A**	0.03 (-0.21, 0.27)	0.26 (-0.16, 0.68)	0.05 (-0.37, 0.47)	0.41	0.93	0.99	1.00
rs8038920	G/**A**	0.08 (-0.17, 0.32)	-0.19 (-0.63, 0.25)	-0.27 (-0.71, 0.16)	0.30	0.13	0.99	0.93
rs4887077	**A**/G	-0.06 (-0.3, 0.17)	-0.30 (-0.71, 0.11)	-0.39 (-0.79, 0.01)	0.40	0.19	0.99	0.98
rs11638372	**A**/G	-0.07 (-0.3, 0.17)	-0.30 (-0.71, 0.11)	-0.40 (-0.80, 0.00)	0.41	0.19	0.99	0.97

[a] Effect allele is the smoking-increasing allele. Minor allele is in bold.
[b] Linear regression model including SNP, three first PCs.
[c] Interaction model including SNP, gender, smoking (no, light, heavy), three first PCs, SNP*smoking. The interaction terms are for SNP*light smoking (A) and SNP*heavy smoking (B).
[d] Adjustment for multiple testing by MaxT bootstrap test for gene-environment interaction.
