# Supplementary material for: Associations between Variation in CHRNA5-CHRNA3-CHRNB4, Body Mass Index and Blood Pressure in the Northern Finland Birth Cohort 1966
Source: PLoS One. 2012 Sep 27;7(9):e46557. doi: 10.1371/journal.pone.0046557 (PMC3459914; doi:10.1371/journal.pone.0046557)
Supplement: Table S4 — Estimated associations between variants in the 15q25 region and SBP according to smoking status (never, former and current smokers) in NFBC1966. (PDF) [file pone.0046557.s004.pdf]

Table S4. Estimated associations between variants in the 15q25 region and SBP according to smoking status (never, former and current smokers) in the NFBC1966.

		Never smokers (N=1813-1822)	Former smokers (N=945-949)	Current smokers (N=2028-2042)				
**rs number**	**Effect/ other allele** [a]	**beta (95% CI)** [b]	**beta (95% CI)** [b]	**beta (95% CI)** [b]	***P*-value for interaction (A)** [c]	***P*-value for interaction (B)** [c]	**Adjusted *P*-value for interaction (A)** [d]	**Adjusted *P*-value for interaction (B)** [d]
rs8034191	**G**/A	-0.04 (-0.92, 0.84)	-0.11 (-1.23, 1.00)	-0.21 (-0.99, 0.56)	0.98	0.73	1.00	1.00
rs3885951	**G**/A	0.19 (-1.54, 1.92)	0.68 (-1.57, 2.94)	-0.43 (-1.93, 1.07)	0.72	0.56	1.00	1.00
rs2036534	A/**G**	-0.12 (-1.04, 0.79)	0.23 (-0.93, 1.39)	0.43 (-0.40, 1.25)	0.66	0.40	1.00	0.99
rs6495306	A/**G**	0.05 (-0.80, 0.91)	-0.57 (-1.63, 0.49)	-0.58 (-1.34, 0.18)	0.42	0.25	1.00	0.99
rs680244	G/**A**	0.04 (-0.81, 0.90)	-0.54 (-1.60, 0.52)	-0.56 (-1.32, 0.20)	0.45	0.28	1.00	0.99
rs621849	A/**G**	0.02 (-0.84, 0.87)	-0.53 (-1.59, 0.53)	-0.57 (-1.33, 0.19)	0.47	0.29	1.00	0.99
rs1051730	**A**/G	-0.10 (-0.98, 0.79)	-0.19 (-1.32, 0.94)	-0.60 (-1.38, 0.18)	0.93	0.35	1.00	0.99
rs6495309	G/**A**	-0.18 (-1.10, 0.74)	0.69 (-0.48, 1.86)	0.18 (-0.66, 1.02)	0.27	0.59	0.99	1.00
rs1948	G/**A**	-0.02 (-0.88, 0.84)	-0.50 (-1.57, 0.57)	-1.24 (-2.03, -0.45)	0.50	0.03	1.00	0.45
rs950776	A/**G**	0.00 (-0.87, 0.87)	-0.43 (-1.53, 0.67)	-1.17 (-1.97, -0.37)	0.65	0.04	1.00	0.56
rs12594247	**A**/G	0.36 (-0.67, 1.39)	-0.70 (-1.99, 0.60)	-0.56 (-1.47, 0.35)	0.28	0.18	0.99	0.97
rs12900519	A/**G**	-0.01 (-1.16, 1.15)	-1.58 (-3.04, -0.13)	-0.49 (-1.57, 0.59)	0.10	0.49	0.86	1.00
rs1996371	**G**/A	-0.34 (-1.22, 0.54)	-0.35 (-1.47, 0.78)	-0.23 (-1.00, 0.55)	0.93	0.93	1.00	1.00
rs6495314	**C**/A	-0.42 (-1.30, 0.46)	-0.43 (-1.56, 0.70)	-0.25 (-1.02, 0.52)	0.94	0.85	1.00	1.00
rs8032156	G/**A**	0.02 (-0.89, 0.94)	0.92 (-0.20, 2.04)	0.11 (-0.69, 0.91)	0.19	0.82	0.98	1.00
rs8038920	G/**A**	-0.31 (-1.25, 0.62)	-0.27 (-1.44, 0.90)	-0.52 (-1.36, 0.32)	0.75	0.71	1.00	1.00
rs4887077	**A**/G	-0.30 (-1.18, 0.59)	-0.55 (-1.69, 0.60)	-0.17 (-0.96, 0.61)	0.82	0.92	1.00	1.00
rs11638372	**A**/G	-0.24 (-1.12, 0.65)	-0.55 (-1.69, 0.60)	-0.17 (-0.95, 0.62)	0.76	0.99	1.00	1.00

[a] Effect allele is the smoking-increasing allele. Minor allele is in bold.
[b] Linear regression model including SNP, gender, BMI at 31 years, three first PCs.
[c] Interaction model including SNP, gender, BMI at 31 years, smoking (never, former, current smoker), three first PCs, SNP*smoking. The interaction terms are for SNP*former smoker (A) and SNP*current smoker (B).
[d] Adjustment for multiple testing by MaxT bootstrap test for gene-environment interaction.
