# Supplementary material for: Associations between Variation in CHRNA5-CHRNA3-CHRNB4, Body Mass Index and Blood Pressure in the Northern Finland Birth Cohort 1966
Source: PLoS One. 2012 Sep 27;7(9):e46557. doi: 10.1371/journal.pone.0046557 (PMC3459914; doi:10.1371/journal.pone.0046557)
Supplement: Table S5 — Estimated associations between variants in the 15q25 region and DBP according to smoking status (never, former and current smokers) in the NFBC1966. (PDF) [file pone.0046557.s005.pdf]

Table S5. Estimated associations between variants in the 15q25 region and DBP according to smoking status (never, former and current smokers) in the NFBC1966.

		Never smokers (N=1811-1820)	Former smokers (N=946-948)	Current smokers (N=2025-2039)				
**rs number**	**Effect/ other allele [a]**	**beta (95% CI) [b]**	**beta (95% CI) [b]**	**beta (95% CI) [b]**	***P*-value for interaction (A) [c]**	***P*-value for interaction (B) [c]**	**Adjusted *P*-value for interaction (A) [d]**	**Adjusted *P*-value for interaction (B) [d]**
rs8034191	**G**/A	0.18 (-0.57, 0.93)	-0.12 (-1.12, 0.89)	-0.14 (-0.83, 0.54)	0.70	0.51	1.00	1.00
rs3885951	**G**/A	0.41 (-1.06, 1.89)	-0.52 (-2.55, 1.51)	0.14 (-1.18, 1.46)	0.52	0.78	1.00	1.00
rs2036534	A/**G**	0.59 (-0.19, 1.37)	0.15 (-0.89, 1.19)	-0.12 (-0.85, 0.6)	0.51	0.18	1.00	0.97
rs6495306	A/**G**	-0.33 (-1.06, 0.40)	-0.42 (-1.37, 0.54)	-0.09 (-0.76, 0.58)	0.96	0.64	1.00	1.00
rs680244	G/**A**	-0.35 (-1.08, 0.38)	-0.42 (-1.37, 0.54)	-0.08 (-0.74, 0.59)	0.98	0.60	1.00	1.00
rs621849	A/**G**	-0.36 (-1.09, 0.37)	-0.43 (-1.38, 0.52)	-0.09 (-0.76, 0.58)	0.98	0.59	1.00	1.00
rs1051730	**A**/G	0.12 (-0.64, 0.87)	-0.04 (-1.06, 0.97)	-0.27 (-0.96, 0.42)	0.85	0.41	1.00	1.00
rs6495309	G/**A**	0.42 (-0.36, 1.21)	0.31 (-0.74, 1.36)	-0.39 (-1.13, 0.34)	0.84	0.13	1.00	0.91
rs1948	G/**A**	-0.29 (-1.03, 0.44)	-0.29 (-1.25, 0.68)	-0.09 (-0.78, 0.61)	0.94	0.72	1.00	1.00
rs950776	A/**G**	-0.42 (-1.16, 0.33)	-0.35 (-1.34, 0.65)	-0.14 (-0.84, 0.57)	0.80	0.60	1.00	1.00
rs12594247	**A**/G	-0.19 (-1.06, 0.69)	-0.38 (-1.55, 0.79)	-0.24 (-1.04, 0.56)	0.91	0.94	1.00	1.00
rs12900519	A/**G**	0.44 (-0.55, 1.43)	-1.18 (-2.49, 0.13)	0.58 (-0.37, 1.53)	0.06	0.93	0.70	1.00
rs1996371	**G**/A	0.02 (-0.73, 0.77)	-0.85 (-1.86, 0.16)	-0.02 (-0.70, 0.66)	0.22	0.86	0.99	1.00
rs6495314	**C**/A	-0.05 (-0.80, 0.70)	-0.85 (-1.86, 0.16)	-0.04 (-0.72, 0.65)	0.26	0.94	0.99	1.00
rs8032156	G/**A**	-0.50 (-1.28, 0.28)	0.60 (-0.40, 1.61)	-0.57 (-1.27, 0.13)	0.08	1.00	0.80	1.00
rs8038920	G/**A**	-0.39 (-1.19, 0.41)	-0.77 (-1.82, 0.28)	-0.73 (-1.47, 0.01)	0.73	0.55	1.00	1.00
rs4887077	**A**/G	0.08 (-0.68, 0.84)	-1.14 (-2.17, -0.11)	-0.04 (-0.73, 0.65)	0.09	0.75	0.80	1.00
rs11638372	**A**/G	0.09 (-0.66, 0.85)	-1.14 (-2.17, -0.11)	-0.05 (-0.74, 0.65)	0.08	0.73	0.79	1.00

[a] Effect allele is the smoking-increasing allele. Minor allele is in bold.
[b] Linear regression model including SNP, gender, BMI at 31 years, three first PCs.
[c] Interaction model including SNP, gender, BMI at 31 years, smoking (never, former, current smokers), three first PCs, SNP*smoking. The interaction terms are for SNP*former smoker (A) and SNP*current smoker (B).
[d] Adjustment for multiple testing by MaxT bootstrap test for gene-environment interaction.
