# Supplementary material for: Associations between Variation in CHRNA5-CHRNA3-CHRNB4, Body Mass Index and Blood Pressure in the Northern Finland Birth Cohort 1966
Source: PLoS One. 2012 Sep 27;7(9):e46557. doi: 10.1371/journal.pone.0046557 (PMC3459914; doi:10.1371/journal.pone.0046557)
Supplement: Table S6 — Estimated associations between variants in the 15q25 region and BMI according to smoking status (never, former and current smokers) in the NFBC1966. (PDF) [file pone.0046557.s006.pdf]

Table S6. Estimated associations between variants in the 15q25 region and BMI according to smoking status (never, former and current smokers) in the NFBC1966.

		Never smokers (N=1813-1822)	Former smokers (N=945-949)	Current smokers (N=2028-2042)				
**rs number**	**Effect/ other allele [a]**	**beta (95% CI) [b]**	**beta (95% CI) [b]**	**beta (95% CI) [b]**	***P*-value for interaction (A) [c]**	***P*-value for interaction (B) [c]**	**Adjusted *P*-value for interaction (A) [d]**	**Adjusted *P*-value for interaction (B) [d]**
rs8034191	**G**/A	0.05 (-0.24, 0.34)	-0.04 (-0.43, 0.35)	-0.26 (-0.54, 0.03)	0.79	0.15	1.00	0.95
rs3885951	**G**/A	-0.24 (-0.80, 0.33)	0.22 (-0.57, 1.00)	0.09 (-0.46, 0.64)	0.35	0.37	0.99	0.99
rs2036534	A/**G**	0.11 (-0.19, 0.41)	-0.23 (-0.64, 0.17)	-0.35 (-0.65, -0.05)	0.20	0.04	0.99	0.48
rs6495306	A/**G**	0.01 (-0.27, 0.29)	0.07 (-0.30, 0.44)	0.07 (-0.21, 0.35)	0.74	0.74	1.00	1.00
rs680244	G/**A**	0.02 (-0.26, 0.30)	0.09 (-0.28, 0.45)	0.07 (-0.21, 0.35)	0.72	0.75	1.00	1.00
rs621849	A/**G**	0.02 (-0.26, 0.30)	0.08 (-0.29, 0.45)	0.07 (-0.21, 0.35)	0.74	0.76	1.00	1.00
rs1051730	**A**/G	0.09 (-0.20, 0.38)	-0.09 (-0.48, 0.31)	-0.24 (-0.53, 0.04)	0.57	0.14	1.00	0.93
rs6495309	G/**A**	0.01 (-0.29, 0.31)	-0.12 (-0.52, 0.29)	-0.44 (-0.75, -0.14)	0.63	0.04	1.00	0.48
rs1948	G/**A**	0.04 (-0.24, 0.33)	-0.07 (-0.45, 0.30)	-0.02 (-0.31, 0.27)	0.71	0.83	1.00	1.00
rs950776	A/**G**	0.03 (-0.25, 0.32)	-0.12 (-0.50, 0.27)	0.02 (-0.28, 0.31)	0.65	0.99	1.00	1.00
rs12594247	**A**/G	0.17 (-0.17, 0.51)	-0.22 (-0.67, 0.23)	0.24 (-0.10, 0.57)	0.23	0.78	0.99	1.00
rs12900519	A/**G**	-0.10 (-0.48, 0.28)	-0.35 (-0.86, 0.16)	0.11 (-0.28, 0.51)	0.53	0.33	1.00	0.99
rs1996371	**G**/A	0.08 (-0.21, 0.37)	-0.23 (-0.62, 0.16)	-0.41 (-0.70, -0.13)	0.27	0.02	0.99	0.33
rs6495314	**C**/A	0.08 (-0.21, 0.36)	-0.20 (-0.59, 0.19)	-0.41 (-0.69, -0.12)	0.33	0.02	0.99	0.37
rs8032156	G/**A**	-0.06 (-0.36, 0.24)	0.16 (-0.23, 0.55)	0.15 (-0.15, 0.45)	0.42	0.42	1.00	1.00
rs8038920	G/**A**	0.23 (-0.08, 0.53)	-0.21 (-0.62, 0.19)	-0.22 (-0.53, 0.09)	0.12	0.04	0.91	0.48
rs4887077	**A**/G	0.02 (-0.27, 0.31)	-0.22 (-0.61, 0.18)	-0.33 (-0.62, -0.05)	0.43	0.12	1.00	0.91
rs11638372	**A**/G	0.01 (-0.28, 0.30)	-0.22 (-0.61, 0.18)	-0.34 (-0.62, -0.05)	0.44	0.12	1.00	0.91

[a] Effect allele is the smoking-increasing allele. Minor allele is in bold.
[b] Linear regression model including SNP, three first PCs.
[c] Interaction model including SNP, gender, BMI at 31 years, smoking (never, former, current smoker), three first PCs, SNP*smoking. The interaction terms are for SNP*former smoker (A) and SNP*current smoker (B).
[d] Adjustment for multiple testing by MaxT bootstrap test for gene-environment interaction.
