# Supplementary material for: Associations between Variation in CHRNA5-CHRNA3-CHRNB4, Body Mass Index and Blood Pressure in the Northern Finland Birth Cohort 1966
Source: PLoS One. 2012 Sep 27;7(9):e46557. doi: 10.1371/journal.pone.0046557 (PMC3459914; doi:10.1371/journal.pone.0046557)
Supplement: Table S7 — Estimated associations between variants in the 15q25 region and SBP according to gender in the NFBC1966. (PDF) [file pone.0046557.s007.pdf]

Table S7. Estimated associations between variants in the 15q25 region and SBP according to gender in the NFBC1966.

		**Males (N=2327-2338)**	**Females (N=2459-2475)**		
**rs number**	**Effect/ other allele** [a]	**beta (95% CI)** [b]	**beta (95% CI)** [b]	***P*-value for interaction** [c]	**Adjusted *P*-value for interaction** [d]
rs8034191	**G**/A	-0.08 (-0.84, 0.67)	-0.15 (-0.86, 0.56)	0.96	1.00
rs3885951	**G**/A	-0.33 (-1.80, 1.15)	0.27 (-1.13, 1.66)	0.56	1.00
rs2036534	A/**G**	-0.20 (-1.00, 0.59)	0.53 (-0.21, 1.27)	0.16	0.95
rs6495306	A/**G**	-0.06 (-0.78, 0.67)	-0.58 (-1.27, 0.12)	0.30	0.99
rs680244	G/**A**	-0.04 (-0.77, 0.68)	-0.57 (-1.26, 0.13)	0.30	0.99
rs621849	A/**G**	-0.04 (-0.77, 0.68)	-0.59 (-1.29, 0.10)	0.27	0.99
rs1051730	**A**/G	-0.4 (-1.17, 0.36)	-0.25 (-0.96, 0.47)	0.72	1.00
rs6495309	G/**A**	-0.09 (-0.89, 0.72)	0.33 (-0.42, 1.08)	0.39	1.00
rs1948	G/**A**	-0.26 (-1.00, 0.48)	-0.92 (-1.63, -0.21)	0.20	0.99
rs950776	A/**G**	-0.28 (-1.03, 0.47)	-0.80 (-1.53, -0.08)	0.33	0.99
rs12594247	**A**/G	0.11 (-0.78, 1.00)	-0.56 (-1.39, 0.26)	0.26	0.99
rs12900519	A/**G**	-0.03 (-1.02, 0.96)	-1.06 (-2.04, -0.08)	0.13	0.93
rs1996371	**G**/A	-0.11 (-0.87, 0.65)	-0.37 (-1.08, 0.33)	0.69	1.00
rs6495314	**C**/A	-0.18 (-0.94, 0.58)	-0.41 (-1.12, 0.29)	0.73	1.00
rs8032156	G/**A**	-0.16 (-0.92, 0.61)	0.59 (-0.15, 1.34)	0.16	0.97
rs8038920	G/**A**	-0.19 (-1.00, 0.61)	-0.50 (-1.26, 0.26)	0.70	1.00
rs4887077	**A**/G	-0.06 (-0.83, 0.71)	-0.41 (-1.12, 0.31)	0.58	1.00
rs11638372	**A**/G	-0.03 (-0.80, 0.73)	-0.38 (-1.10, 0.33)	0.59	1.00

[a] Effect allele is the smoking-increasing allele. Minor allele is in bold.
[b] Linear regression model including SNP, gender, BMI at 31 years, smoking (no, light, heavy), three first PCs.
[c] Interaction model including SNP, gender, BMI at 31 years, smoking (no, light, heavy), three first PCs, SNP*gender.
[d] Adjustment for multiple testing by MaxT bootstrap test for gene-environment interaction.
