# Supplementary material for: Associations between Variation in CHRNA5-CHRNA3-CHRNB4, Body Mass Index and Blood Pressure in the Northern Finland Birth Cohort 1966
Source: PLoS One. 2012 Sep 27;7(9):e46557. doi: 10.1371/journal.pone.0046557 (PMC3459914; doi:10.1371/journal.pone.0046557)
Supplement: Table S8 — Estimated associations between variants in the 15q25 region and DBP according to gender in NFBC1966. (PDF) [file pone.0046557.s008.pdf]

Table S8. Estimated associations between variants in the 15q25 region and DBP according to gender in the NFBC1966.

		Males (N=2327-2338)	Females (N=2453-2469)		
**rs number**	**Effect/ other allele [a]**	**beta (95% CI) [b]**	**beta (95% CI) [b]**	***P*-value for interaction [c]**	**Adjusted *P*-value for interaction [d]**
rs8034191	**G**/A	-0.07 (-0.73, 0.60)	0.07 (-0.54, 0.69)	0.64	1.00
rs3885951	**G**/A	-0.79 (-2.09, 0.50)	0.93 (-0.27, 2.13)	0.05	0.56
rs2036534	A/**G**	-0.41 (-1.11, 0.29)	0.78 (0.15, 1.42)	0.01	0.15
rs6495306	A/**G**	0.18 (-0.46, 0.82)	-0.61 (-1.21, -0.01)	0.09	0.80
rs680244	G/**A**	0.18 (-0.46, 0.82)	-0.61 (-1.21, -0.02)	0.09	0.79
rs621849	A/**G**	0.18 (-0.46, 0.82)	-0.64 (-1.24, -0.04)	0.08	0.74
rs1051730	**A**/G	-0.20 (-0.87, 0.47)	0.07 (-0.54, 0.69)	0.44	1.00
rs6495309	G/**A**	-0.60 (-1.31, 0.11)	0.67 (0.02, 1.31)	0.01	0.11
rs1948	G/**A**	0.36 (-0.30, 1.01)	-0.68 (-1.29, -0.07)	0.03	0.40
rs950776	A/**G**	0.17 (-0.49, 0.83)	-0.65 (-1.27, -0.03)	0.10	0.84
rs12594247	**A**/G	0.31 (-0.48, 1.09)	-0.73 (-1.44, -0.02)	0.05	0.58
rs12900519	A/**G**	0.45 (-0.42, 1.32)	-0.16 (-1.01, 0.68)	0.34	0.99
rs1996371	**G**/A	-0.34 (-1.01, 0.32)	0.09 (-0.52, 0.70)	0.24	0.99
rs6495314	**C**/A	-0.39 (-1.06, 0.28)	0.07 (-0.54, 0.68)	0.21	0.99
rs8032156	G/**A**	-0.48 (-1.16, 0.19)	-0.15 (-0.79, 0.48)	0.54	1.00
rs8038920	G/**A**	-0.60 (-1.31, 0.10)	-0.51 (-1.17, 0.14)	0.74	1.00
rs4887077	**A**/G	-0.38 (-1.06, 0.29)	0.05 (-0.56, 0.67)	0.24	0.99
rs11638372	**A**/G	-0.39 (-1.06, 0.29)	0.06 (-0.55, 0.68)	0.23	0.99

[a] Effect allele is the smoking-increasing allele. Minor allele is in bold.
[b] Linear regression model including SNP, gender, BMI at 31 years, smoking (no, light, heavy), three first PCs.
[c] Interaction model including SNP, gender, BMI at 31 years, smoking (no, light, heavy), three first PCs, SNP*gender.
[d] Adjustment for multiple testing by MaxT bootstrap test for gene-environment interaction.
