# Supplementary material for: Associations between Variation in CHRNA5-CHRNA3-CHRNB4, Body Mass Index and Blood Pressure in the Northern Finland Birth Cohort 1966
Source: PLoS One. 2012 Sep 27;7(9):e46557. doi: 10.1371/journal.pone.0046557 (PMC3459914; doi:10.1371/journal.pone.0046557)
Supplement: Table S9 — Estimated associations between variants in the 15q25 region and BMI according to gender in the NFBC1966. (PDF) [file pone.0046557.s009.pdf]

Table S9. Estimated associations between variants in the 15q25 region and BMI according to gender in the NFBC1966.

		Males (N=2489-2502)	Females (N=2604-2623)		
**rs number**	**Effect/ other allele** [a]	**beta (95% CI)** [b]	**beta (95% CI)** [b]	***P*-value for interaction** [c]	**Adjusted *P*-value for interaction** [d]
rs8034191	**G**/A	-0.18 (-0.39, 0.03)	0.00 (-0.27, 0.28)	0.49	1.00
rs3885951	**G**/A	0.05 (-0.36, 0.46)	-0.06 (-0.59, 0.47)	0.85	1.00
rs2036534	A/**G**	-0.22 (-0.45, 0.00)	-0.05 (-0.34, 0.23)	0.48	1.00
rs6495306	A/**G**	0.00 (-0.21, 0.20)	0.10 (-0.16, 0.37)	0.60	1.00
rs680244	G/**A**	0.00 (-0.20, 0.20)	0.11 (-0.16, 0.38)	0.58	1.00
rs621849	A/**G**	0.00 (-0.21, 0.20)	0.11 (-0.16, 0.37)	0.59	1.00
rs1051730	**A**/G	-0.18 (-0.39, 0.04)	0.03 (-0.25, 0.30)	0.40	0.99
rs6495309	G/**A**	-0.25 (-0.48, -0.03)	-0.11 (-0.40, 0.17)	0.65	1.00
rs1948	G/**A**	-0.10 (-0.31, 0.11)	0.10 (-0.18, 0.37)	0.22	0.99
rs950776	A/**G**	-0.08 (-0.29, 0.13)	0.08 (-0.20, 0.36)	0.35	0.99
rs12594247	**A**/G	0.17 (-0.08, 0.42)	0.09 (-0.22, 0.41)	0.75	1.00
rs12900519	A/**G**	0.10 (-0.18, 0.38)	-0.26 (-0.64, 0.12)	0.15	0.94
rs1996371	**G**/A	-0.29 (-0.51, -0.08)	-0.11 (-0.38, 0.16)	0.39	0.99
rs6495314	**C**/A	-0.29 (-0.50, -0.08)	-0.10 (-0.37, 0.17)	0.38	0.99
rs8032156	G/**A**	0.04 (-0.17, 0.26)	0.14 (-0.14, 0.42)	0.67	1.00
rs8038920	G/**A**	-0.22 (-0.44, 0.01)	0.12 (-0.17, 0.42)	0.11	0.88
rs4887077	**A**/G	-0.30 (-0.51, -0.08)	-0.07 (-0.34, 0.21)	0.27	0.99
rs11638372	**A**/G	-0.30 (-0.51, -0.08)	-0.07 (-0.35, 0.20)	0.29	0.99

[a] Effect allele is the smoking-increasing allele. Minor allele is in bold.

[b] Linear regression model including SNP, gender, BMI at 31 years, smoking (no, light, heavy), three first PCs.

[c] Interaction model including SNP, gender, BMI at 31 years, smoking (no, light, heavy), three first PCs, SNP*gender.

[d] Adjustment for multiple testing by MaxT bootstrap test for gene-environment interaction.
