# Supplementary material for: Associations between Variation in CHRNA5-CHRNA3-CHRNB4, Body Mass Index and Blood Pressure in the Northern Finland Birth Cohort 1966
Source: PLoS One. 2012 Sep 27;7(9):e46557. doi: 10.1371/journal.pone.0046557 (PMC3459914; doi:10.1371/journal.pone.0046557)
Supplement: Table S10 — Estimated haplotype frequencies and estimates from association analyses (Block 1) for SBP and BMI in smokers in the NFBC1966. (PDF) [file pone.0046557.s010.pdf]

Table S10. Estimated haplotype frequencies and estimates from association analyses (Block 1) for SBP and BMI in smokers in the NFBC1966.

Outcome	rs8034191	rs3885951	rs2036534	rs6495306	rs680244	rs621849	rs1051730	rs6495309	rs1948	rs950776	Frequency [a]	Beta (95% CI) [b]	*P*-value [c]	Adjusted *P*-value [d]
SBP														
					G	A	G	G	A	G	0.01	6.16 (2.58, 9.74)	$7.3x10^{-4}$	0.02
						A	G	G	A	G	0.01	6.15 (2.58, 9.72)	$7.5x10^{-4}$	0.02
				A	G	A	G	G	A	G	0.01	6.14 (2.58, 9.70)	$7.6x10^{-4}$	0.02
					G	A	G	G	A		0.01	5.98 (2.41, 9.55)	0.001	0.02
				A	G	A	G	G	A		0.01	5.98 (2.41, 9.55)	0.001	0.02
						A	G	G	A		0.01	5.97 (2.41, 9.53)	0.001	0.02
		A	A	A	G	A	G				0.03	3.76 (1.24, 6.28)	0.004	0.05
					A	G	G	G	A		0.03	3.71 (1.21, 6.21)	0.004	0.06
								G	A		0.34	1.24 (0.45, 2.03)	0.002	0.04
							G	C	A		0.34	1.23 (0.44, 2.02)	0.002	0.04
									A	G	0.31	1.19 (0.38, 2.00)	0.004	0.06
								G	A	G	0.31	1.19 (0.38, 2.00)	0.004	0.06
							G	G	A	G	0.31	1.18 (0.37, 1.99)	0.004	0.07
						G	G	G	A		0.33	0.95 (0.15, 1.75)	0.02	0.26
			A	A	A	G	A	G			0.33	0.95 (0.15, 1.75)	0.02	0.26
				G	A	G	G	G	A		0.33	0.95 (0.15, 1.75)	0.02	0.26
			A	G	A	G	G	G	A		0.32	0.95 (0.15, 1.75)	0.02	0.26
		A	A	G	A	G	G	G	A		0.33	0.95 (0.15, 1.75)	0.02	0.26
			A	G	A	G	G	G	A	G	0.30	0.89 (0.07, 1.71)	0.03	0.36
	A	A	A	G	A	G	G	G	A		0.32	0.89 (0.09, 1.69)	0.03	0.38
						G	G	G	A	G	0.30	0.88 (0.07, 1.69)	0.03	0.38
					A	G	G	G	A	G	0.30	0.88 (0.07, 1.69)	0.03	0.39
				G	A	G	G	G	A	G	0.30	0.88 (0.06, 1.70)	0.03	0.39
		A	A	G	A	G	G	G	A	G	0.30	0.88 (0.07, 1.69)	0.03	0.39
	A	A	A	G	A	G	G	G	A	G	0.30	0.83 (0.01, 1.65)	0.05	0.49
								G	G		0.39	-0.99 (-1.75, -0.23)	0.01	0.13
								G	G	A	0.38	-1.02 (-1.78, -0.26)	0.01	0.11
									G	A	0.65	-1.23 (-2.02, -0.44)	0.002	0.04
							G	G	G		0.07	-1.44 (-2.84, -0.04)	0.04	0.52
							G	G	G	A	0.06	-1.82 (-3.36, -0.28)	0.02	0.30
						G	G	G	G	A	0.04	-1.92 (-3.79, -0.05)	0.04	0.49
					A	G	G	G	G	A	0.04	-1.92 (-3.79, -0.05)	0.04	0.49

Outcome	rs8034191	rs3885951	rs2036534	rs6495306	rs680244	rs621849	rs1051730	rs6495309	rs1948	rs950776	Frequency [a]	Beta (95% CI) [b]	*P*-value [c]	Adjusted *P*-value [d]
			A	G	A	G	G	G	G	A	0.04	-1.94 (-3.81, -0.07)	0.04	0.48
	A	A	A	G	A	G	G	G	G	A	0.04	-2.13 (-4.04, -0.22)	0.03	0.36
BMI														
								A	G		0.27	0.45 (0.14, 0.76)	0.004	0.10
						A	G	A			0.27	0.45 (0.14, 0.76)	0.004	0.10
							G	A	G		0.27	0.45 (0.14, 0.76)	0.004	0.10
					G	A	G	A			0.27	0.45 (0.14, 0.76)	0.004	0.10
						A	G	A	G		0.27	0.45 (0.14, 0.76)	0.004	0.10
				A	G	A	G	A			0.27	0.45 (0.14, 0.76)	0.004	0.10
					G	A	G	A	G		0.27	0.45 (0.14, 0.76)	0.004	0.10
				A	G	A	G	A	G		0.27	0.45 (0.14, 0.76)	0.004	0.10
							G	A			0.27	0.44 (0.14, 0.74)	0.004	0.10
			G	A	G	A	G	A			0.25	0.43 (0.12, 0.74)	0.01	0.14
		A	G	A	G	A	G	A			0.25	0.43 (0.12, 0.74)	0.01	0.14
			G	A	G	A	G	A	G		0.25	0.43 (0.12, 0.74)	0.01	0.14
	A	A	G	A	G	A	G	A			0.25	0.43 (0.12, 0.74)	0.01	0.15
		A	G	A	G	A	G	A	G		0.25	0.43 (0.12, 0.74)	0.01	0.15
	A	A	G	A	G	A	G	A	G		0.25	0.43 (0.12, 0.74)	0.01	0.15
								A	G	A	0.27	0.42 (0.11, 0.73)	0.01	0.14
							G	A	G	A	0.27	0.42 (0.11, 0.73)	0.01	0.15
						A	G	A	G	A	0.27	0.42 (0.11, 0.73)	0.01	0.15
					G	A	G	A	G	A	0.27	0.42 (0.11, 0.73)	0.01	0.15
				A	G	A	G	A	G	A	0.27	0.42 (0.12, 0.72)	0.01	0.16
			G	A	G	A	G	A	G	A	0.25	0.40 (0.09, 0.71)	0.01	0.22
		A	G	A	G	A	G	A	G	A	0.25	0.40 (0.09, 0.71)	0.01	0.23
	A	A	G	A	G	A	G	A	G	A	0.25	0.40 (0.09, 0.71)	0.01	0.23
		A	G								0.28	0.35 (0.05, 0.65)	0.02	0.36
			G	A							0.28	0.35 (0.04, 0.66)	0.03	0.36
	A	A	G								0.28	0.35 (0.04, 0.66)	0.03	0.36
		A	G	A							0.28	0.35 (0.05, 0.65)	0.02	0.36
			G	A	G						0.28	0.35 (0.05, 0.65)	0.02	0.36
	A	A	G	A							0.28	0.35 (0.05, 0.65)	0.02	0.36
		A	G	A	G						0.28	0.35 (0.05, 0.65)	0.02	0.36
			G	A	G	A					0.28	0.35 (0.05, 0.65)	0.02	0.36
	A	A	G	A	G						0.28	0.35 (0.05, 0.65)	0.02	0.37

Outcome	rs8034191	rs3885951	rs2036534	rs6495306	rs680244	rs621849	rs1051730	rs6495309	rs1948	rs950776	Frequency [a]	Beta (95% CI) [b]	*P*-value [c]	Adjusted *P*-value [d]
		A	G	A	G	A					0.28	0.35 (0.05, 0.65)	0.02	0.37
			G	A	G	A	G				0.28	0.35 (0.05, 0.65)	0.02	0.37
	A	A	G	A	G	A					0.28	0.35 (0.05, 0.65)	0.02	0.37
		A	G	A	G	A	G				0.28	0.35 (0.05, 0.65)	0.02	0.39
	A	A	G	A	G	A	G				0.28	0.35 (0.05, 0.65)	0.02	0.38
					G	A	G				0.30	0.34 (0.04, 0.64)	0.03	0.41
				A	G	A	G				0.30	0.34 (0.04, 0.64)	0.03	0.41
						A	G				0.30	0.33 (0.04, 0.62)	0.03	0.41
	G	A	A	A							0.26	-0.30 (-0.60, 0.00)	0.05	0.57
	G	A	A	A	G						0.26	-0.30 (-0.60, 0.00)	0.05	0.57
	G	A	A	A	G	A					0.26	-0.30 (-0.60, 0.00)	0.05	0.57
	G	A									0.27	-0.31 (-0.61, -0.01)	0.04	0.52
	G	A	A								0.27	-0.31 (-0.61, -0.01)	0.04	0.51
		A	A								0.66	-0.33 (-0.61, -0.05)	0.02	0.31
								G	G	A	0.38	-0.36 (-0.64, -0.08)	0.01	0.26
								G	G		0.39	-0.39 (-0.67, -0.11)	0.01	0.15
							G	G	G		0.07	-0.52 (-1.04, 0.00)	0.04	0.49

[a] Only haplotypes with frequency >1% were included in the analysis.

[b] The analyses were adjusted for gender, BMI at 31 years (analyses for SBP) and three first PCs.

[c] Associations with P value < 0.05 are presented.

[d] Adjustment for multiple testing by maxT permutation of residuals.
