# Supplementary material for: Associations between Variation in CHRNA5-CHRNA3-CHRNB4, Body Mass Index and Blood Pressure in the Northern Finland Birth Cohort 1966
Source: PLoS One. 2012 Sep 27;7(9):e46557. doi: 10.1371/journal.pone.0046557 (PMC3459914; doi:10.1371/journal.pone.0046557)
Supplement: Table S11 — Estimated haplotype frequencies and estimates from association analyses (Block 2) for DBP and BMI in smokers in the NFBC1966. (PDF) [file pone.0046557.s011.pdf]

Table S11. Estimated haplotype frequencies and estimates from association analyses (Block 2) for DBP and BMI in smokers in the NFBC1966.

Outcome	rs12594247	rs12900519	rs1996371	rs6495314	rs8032156	rs8038920	rs4887077	rs11638372	Frequency [a]	Beta (95% CI) [b]	*P*-Value [c]	Adjusted *P*-value [d]
DBP												
		A	A	A	G	A	G		0.27	0.75 (0.01, 1.49)	0.05	0.92
		A	A	A	G	A			0.27	0.74 (0.00, 1.48)	0.05	1.00
		A	A	A	G	A	G	G	0.27	0.74 (0.00, 1.48)	0.05	0.96
BMI												
	G	A	A	A	A	G			0.02	1.04 (0.07, 2.01)	0.03	0.36
	G	A	A	A	A	G	G		0.02	1.04 (0.08, 2.00)	0.03	0.36
	G	A	A	A	A	G	G	G	0.02	1.04 (0.08, 2.00)	0.03	0.36
	G	A	A	A	A				0.02	1.00 (0.02, 1.98)	0.05	0.42
		A	A						0.51	0.44 (0.17, 0.71)	0.002	0.03
		A	A	A					0.51	0.44 (0.17, 0.71)	0.002	0.03
			A	A					0.65	0.40 (0.12, 0.68)	0.01	0.10
							G	G	0.67	0.34 (0.05, 0.63)	0.02	0.29
	G	A	A						0.30	0.33 (0.03, 0.63)	0.03	0.27
	G	A	A	A					0.30	0.33 (0.04, 0.62)	0.03	0.27
		A	A	A	G				0.35	0.29 (0.01, 0.57)	0.04	0.36
					G	G			0.42	-0.31 (-0.59, -0.03)	0.03	0.33
						G	A		0.33	-0.33 (-0.62, -0.04)	0.03	0.32
						G	A	A	0.33	-0.33 (-0.62, -0.04)	0.02	0.30
				C	G	G	A		0.33	-0.33 (-0.61, -0.05)	0.02	0.30
			G	C	G	G	A		0.33	-0.33 (-0.61, -0.05)	0.02	0.30
				C	G	G	A	A	0.33	-0.33 (-0.61, -0.05)	0.02	0.30
		A	G	C	G	G	A		0.33	-0.33 (-0.61, -0.05)	0.02	0.30
			G	C	G	G	A	A	0.33	-0.33 (-0.61, -0.05)	0.02	0.30
		A	G	C	G	G	A	A	0.33	-0.33 (-0.61, -0.05)	0.02	0.30
							A	A	0.33	-0.34 (-0.63, -0.05)	0.02	0.29
					G	G	A		0.33	-0.34 (-0.63, -0.05)	0.02	0.29
					G	G	A	A	0.33	-0.34 (-0.63, -0.05)	0.02	0.29
	G	A	G	C	G	G	A		0.33	-0.34 (-0.63, -0.05)	0.02	0.29
	G	A	G	C	G	G	A	A	0.33	-0.34 (-0.63, -0.05)	0.02	0.29
			G	C					0.35	-0.41 (-0.70, -0.12)	0.004	0.10
				C	G				0.35	-0.41 (-0.70, -0.12)	0.01	0.09

Outcome	rs12594247	rs12900519	rs1996371	rs6495314	rs8032156	rs8038920	rs4887077	rs11638372	Frequency [a]	Beta (95% CI) [b]	*P*-Value [c]	Adjusted *P*-value [d]
				C	G	G			0.35	-0.41 (-0.69, -0.13)	0.004	0.08
		A	G						0.35	-0.42 (-0.71, -0.13)	0.004	0.08
	G	A	G						0.35	-0.42 (-0.71, -0.13)	0.004	0.08
		A	G	C					0.35	-0.42 (-0.70, -0.14)	0.004	0.08
			G	C	G				0.35	-0.42 (-0.70, -0.14)	0.004	0.08
	G	A	G	C					0.35	-0.42 (-0.70, -0.14)	0.004	0.08
		A	G	C	G				0.35	-0.42 (-0.70, -0.14)	0.004	0.08
			G	C	G	G			0.35	-0.42 (-0.71, -0.13)	0.004	0.08
	G	A	G	C	G				0.35	-0.42 (-0.70, -0.14)	0.004	0.08
		A	G	C	G	G			0.35	-0.42 (-0.71, -0.13)	0.004	0.07
	G	A	G	C	G	G			0.35	-0.42 (-0.71, -0.13)	0.004	0.07
				C	G	G	G		0.02	-1.40 (-2.50, -0.30)	0.01	0.15
			G	C	G	G	G		0.02	-1.40 (-2.50, -0.30)	0.01	0.15
				C	G	G	G	G	0.02	-1.40 (-2.50, -0.30)	0.01	0.15
		A	G	C	G	G	G		0.02	-1.40 (-2.50, -0.30)	0.01	0.15
			G	C	G	G	G	G	0.02	-1.40 (-2.50, -0.30)	0.01	0.15
	G	A	G	C	G	G	G		0.02	-1.40 (-2.50, -0.30)	0.01	0.15
		A	G	C	G	G	G	G	0.02	-1.40 (-2.50, -0.30)	0.01	0.15
	G	A	G	C	G	G	G	G	0.02	-1.40 (-2.50, -0.30)	0.01	0.15

[a] Only haplotypes with frequency >1% were included in the analysis.
[b] The analyses were adjusted for gender, BMI at 31 years (analyses for DBP) and three first PCs.
[c] Associations with *P*-value < 0.05 are presented.
[d] Adjustment for multiple testing by maxT permutation of residuals.
